# Prevalence and associated factors affecting pelvic floor disorder among women in Ethiopia: A systematic review and meta-analysis

**DOI:** 10.1371/journal.pone.0328184

**Published:** 2025-07-18

**Authors:** Tefera Belsty Mihretie, Wubshet Nebiyu Mogess

**Affiliations:** Department of Human Anatomy, School of Medicine, College of Health and Medical Sciences, Haramaya University, Harar, Oromia, Ethiopia; Iran University of Medical Sciences, ISLAMIC REPUBLIC OF IRAN

## Abstract

**Introduction:**

Pelvic floor disorders are a global health problem affecting millions of women, particularly in developing countries. Therefore, this systematic review and meta-analysis aimed to determine the pooled prevalence and common factors affecting pelvic floor disorders among women in Ethiopia.

**Method:**

Databases such as PubMed, Google Scholar, SCOPUS, Hinari and DOAJ were systematically searched to identify eligible studies. The quality of included studies was assessed using Joanna Briggs Institute checklist. Data extraction was performed using Endnote and analyzed using STATA 17 version. To examine possible heterogeneity between studies, the I² test was used. A DerSimonian and Laird random effects model was used to derive pooled prevalence and odds ratio. Funnel plot and Egger’s test were used to detect possible publication bias. This review was registered under PROSPERO ID 2024CRD42024572321.

**Result:**

A total of 8 eligible studies with 9703 participants were included in this systematic review and meta-analysis. The pooled prevalence of pelvic floor disorders among women in Ethiopia was 25% (95% CI 0.20–0.30). Pelvic organ prolapse was the most common pelvic floor disorder 28% (95% CI 0.13–0.43), followed by urinary incontinence 19% (95% CI 0.09–0.29). Vaginal delivery greater than or equal to five (AOR: 4.02.95% CI 1.87–8.79), place of delivery at home (AOR: 1.02.95% CI 0.35–2.98), history of episiotomies (AOR: 2.69.95% CI 0.54–13.49) and women who married under 18 years of age (AOR: 1.32. 95% CI 0.37–4.77) showed a significant association with the prevalence of pelvic floor disorders.

**Conclusion:**

Pelvic organ prolapse is the most common pelvic floor disorder in Ethiopia. Vaginal deliveries, home deliveries, episiotomies and early marriage were identified as factors affecting pelvic floor disorder in Ethiopia.

## Introduction

Pelvic floor dysfunction (PFD) is a disorder characterized by a wide array of clinical situations, which include disorders related to the lower urinary tract, excretion, and defecation, such as urinary and anal incontinence, overactive bladder, pelvic organ prolapses, as well as sexual dysfunction. Among these, pelvic organ prolapse, urinary incontinence, and fecal incontinence are the most prevalent and clinically identifiable conditions that adversely impact the lives of women around the world [1]. PFD is caused by displacement of pelvic organs due to weakness of the tissues supporting the pelvic floor [2]. Pelvic floor disorders are a significant global health problem, particularly in developing countries, affecting millions of people due to limited healthcare access, awareness, and treatment options [3].

All age groups and both genders are affected by pelvic floor dysfunction. [4] with a consistently higher prevalence in older people and women [5].The magnitude of pelvic floor disorders varies from country to country (11.9%−67.5%) [6].

The pelvic floor disorder (PFD) types represent significant health problems for women living in low- and middle-income countries. Prevalence of pelvic organ prolapse (POP) ranging from 3.4% to 56.4%, UI ranging from 5.2% to 70.8%, and the FI is between 5.3% and 41.0% [3,7]. similarly, every, eighth to every fifth woman in Ethiopia suffers from at least one type of pelvic floor disorder, and POP is one of the most common indications for gynecological surgery.[8]. A review of POP was conducted in Ethiopia where 22.70% [9] and 23.52% [10] of patients suffered from POP.

Pelvic floor disorders affect the economic, personal and social aspects of women, in particular their daily activities, sexual life, psychosocial well-being, quality of life and high healthcare costs [11]. Pelvic floor disorders (PFD) can lead to stigmatization in many societies; in turn, women may delay treatment or no seek help at all [12]. In addition, pelvic floor disorders cause impairment and embarrassment for those affected, can lead to social isolation, interfere with task performance, lead to loss of personal and intimate relationships, and reduce participation in recreational activities [13].

Major risk factors for pelvic floor disorders in women include increased parity, vaginal birth, older age, obesity, forceps delivery, prolonged heavy lifting, prolonged second-stage labor, and early age at first delivery [10,14].

There is no published systematic review or meta-analysis on the prevalence of pelvic floor disorders and associated factors in Ethiopia. This review will benefit health care professionals, policymakers and society in developing strategies to prevent and treat pelvic floor dysfunction. This review aims to investigate the pooled prevalence and associated risk factors of PFD in Ethiopia.

### Review question

The review question was designed considering Coco Pop (Condition, Context and Population) as follows “What are the prevalence and associated factors affecting pelvic floor disorder among women in Ethiopia?”.

## Methods and materials

This review followed the guidelines outlined in the Preferred Reporting Items for Systematic Review and Meta-Analysis (PRISMA) checklist.[15] (S1 Table). The systematic review and meta-analysis were duly registered in the International Prospective Register for Systematic Review. PROSPERO2024CRD42024572321Available from: https://www.crd.york.ac.uk/prospero/display_record.php?ID=CRD42024572321

### Eligibility criteria

#### Inclusion criteria.

This review incorporates all observational studies (including case-control, cohort, and cross-sectional designs) conducted in the English language that investigate the prevalence and potential factors influencing pelvic floor disorders in women in Ethiopia. These were considered eligible for inclusion in this systematic review and meta-analysis.

#### Exclusion criteria.

Studies conducted to assess the quality of life of pelvic floor disorder without reporting the prevalence of pelvic floor disorder were excluded. In additionally, unpublished articles were not included in this review. Articles such as letters to the editor, reviews, and articles without complete abstracts or full texts were also excluded from the analysis.

#### Sources of studies and search strategies.

The electronic databases PubMed, Google Scholar, SCOPUS, Hinari and DOAJ were used to access journals and articles published up to 2024. To find all eligible studies in the database, Boolean operator search strategy was used. The key search terms were designed considering condition, context and population using initial keywords (“prevalence” OR “epidemiology” AND “pelvic floor disorder “OR “pelvic floor defect” AND “associated factors” AND “women in Ethiopia”) (S1 File).

#### Quality assessment.

The Joanna Briggs Institute (JBI) standardized critical appraisal checklist was used to assess the quality of individual studies [16]. The checklist contains eight questions which were answered with yes, no, or unclear. Eight cross-sectional studies were evaluated using this checklist, with all articles scoring above half and no articles being rejected. Two authors assessed each article independently, with any discrepancies resolved through discussion and consensus.

### Data extraction

The ENDNOTE citation manager was used to extract data from various sources and remove duplicate articles. Two data extractors were used at different review phases, and any disagreements were resolved through discussion and consensus. Details such as author name, year of publication, study area, study design, sample size, prevalence, cases, and associated factors with adjusted risk ratios (OR) were extracted by two authors (T.B and W.N) and saved in Microsoft Excel.

### Outcome measurement

The primary outcome of this systematic review and meta-analysis was to determine the prevalence of pelvic floor disorders among women in Ethiopia. It was calculated by dividing the sum of pelvic floor disorders by the total sample size and multiplying the result by 100. The secondary outcome included the identification of associated factors influencing pelvic floor disorders, assessed by odds ratio analysis. Factors such as educational status, age, history of vaginal deliveries, home delivery, abortions, participation in weight lifting activities, history of episiotomy, and menopausal status have been recognized as some factors influencing pelvic floor disorders.

### Data analysis

The extracted data was exported to STATA version 17 for further analysis of the pooled prevalence and associated factors affecting pelvic floor disorder among women in Ethiopia. The forest plot and I² heterogeneity test were used to examine the presence of heterogeneity and publication bias within the included studies. A DerSimonian and Laird random effects model was used to derive pooled prevalence and odds ratio. Additionally, a visual inspection of the funnel plot diagram was performed to assess publication bias. Heterogeneity was classified as low, medium, or high based on I² values of 25%, 50%, and 75% respectively [17]. Moreover, meta-regression and sub-group analysis were performed to examine the causes of heterogeneity. Furthermore, to assess the influence of each individual study on the overall estimate, a sensitivity analysis (leave one out) was conducted. Findings were communicated through text descriptions, tabular representations, forest plot diagrams, and effect sizes with 95% confidence intervals.

## Results

### Description of the studies

**Fig 1:**
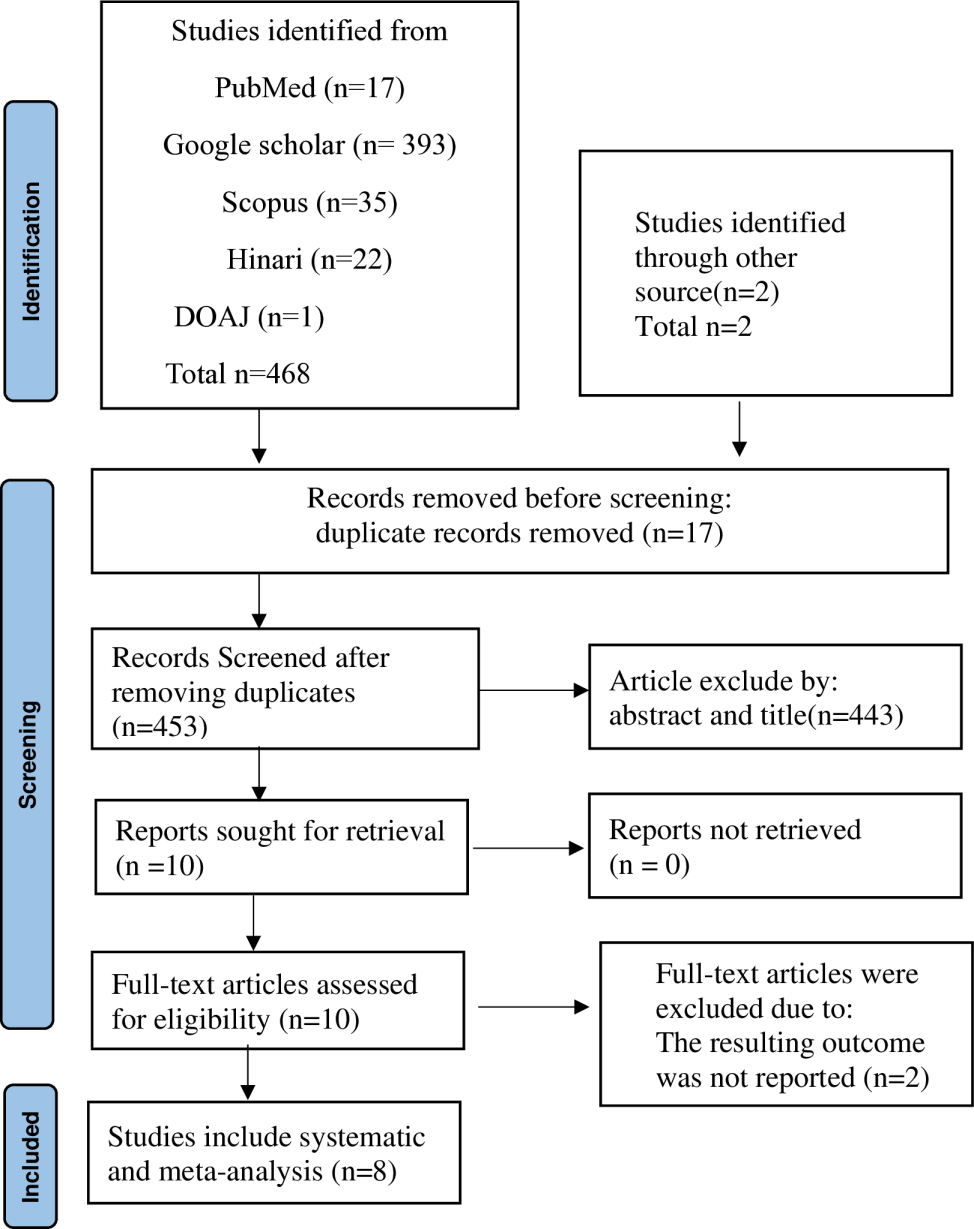
PRISMA flow diagram of the study selection process for systematic review and meta-analysis.

In the present study, a comprehensive search yielded a total of 470 scientific articles from various scientific databases, including PubMed, Google Scholar, DOAJ, Scopus, and the Hinari database. 17 duplicate entries were then systematically removed using ENDNOTES and careful manual review. After removing duplicates, a total of 453 studies were retained, while 443 articles were excluded based on a critical evaluation of their abstracts and titles. A thorough full-text review was performed for 10 articles, 2 of which were rejected due to lack of reported results. Ultimately, the remaining 8 articles that met the predetermined eligibility criteria were included in this systematic review (Fig 1) (S2 Table).

### Quality assessment of included study

All research results were assessed using the Joanna Briggs Institute (JBI) standardized critical appraisal checklist. Based on this checklist, all included papers received scores of over halves, which indicates that they are of high quality and have a minimal bias risk. In the end, eight papers met the criteria set by the Joanna Briggs Institute for a systematic review and meta-analysis.

### Characteristics of the included studies

A total of 8 studies with 9703 participants, including 1549 cases of pelvic floor disorders, were included for a systematic review and meta-analysis. Eight cross-sectional studies [18–25] were used to determine the pooled prevalence of pelvic floor disorders in Ethiopia. Seven cross-sectional studies [18–20,22–25] were used to assess the pooled factors associated with pelvic floor disorder among women in Ethiopia. The publication years of the included studies range from 2013 to 2024 (Table 1).

**Table 1:** Detailed Description of the studies included to analyze the prevalence and contributing factors of pelvic floor disorder among women in Ethiopia: systematic and meta-analysis.

Author	Publication year	Study area	Case	Sample size	Study design	Prevalence	Associated factors	AOR (95%CI)	Quality score 8
Assefa Demissie B, etal [18]	2024	Amhara	59	402	Cross-sectional	14.7	History of heavy loading activities	2.08(0.96-4.53	6
							Place of delivery at home	0.27(0.1-0.73)	
							Has no education	2.16(0.64-7.24	
Beketie ED etal [19]	2021	SNNPR	223	542	Cross-sectional	41.1	History of heavy loading activities	3.38(1.99-5.72)	7
							Vaginal delivery >5	11.18(1.53-81.58)	
							Had abortion history	1.39(0.85-2.26)	
							Menopause	3.37(1.4-8.07)	
							age ≥ 55	0.83(0.16-4.27)	
Benti Terefe A etal [20]	2022	SNNPR	49	275	Cross-sectional	17.8	Vaginal delivery >5	5.193(1.905-14.157)	7
							Episiotomy history	7.508(1.556-36.22)	
							Menopause	7.665(2.44-24.078)	
Dheresa M,etal [22]	2019	Oromia	704	3432	Cross-sectional	20.5	Had abortion history	1.85(1.43-2.38)	8
							Has no education	1.47(1.06-2.04)	
							Episiotomy history	1.39(1.02-1.9)	
Dheresa M,etal [21]	2018	Oromia	704(22)	3432	Cross-sectional	20.5			5
Hambisa HD etal [23]	2023	Benishangul Gumuz	301	798	Cross-sectional	37.7	History of heavy loading activities	3.21(1.86-5.72)	6
							Vaginal delivery >5	4.03(2.2-8.27)	
							Has no education	0.78(0.35-2.09)	
Kebede BN. etal [24]	2023	SNNPR	134	427	Cross-sectional	31.4	Place of delivery at home	1.811(0.905-3.623	6
							Had instrumental delivery	3.042(1.483-6.241	
Megabiaw B etal [25]	2013	Amhara	79	395	Cross-sectional	19.9	History of heavy loading activities	2.302(0.711-7.458	5
							Place of delivery at home	1.811	
							Had instrumental delivery	3.042(1.483-6.241	

### Prevalence of pelvic floor disorder

**Fig 2:**
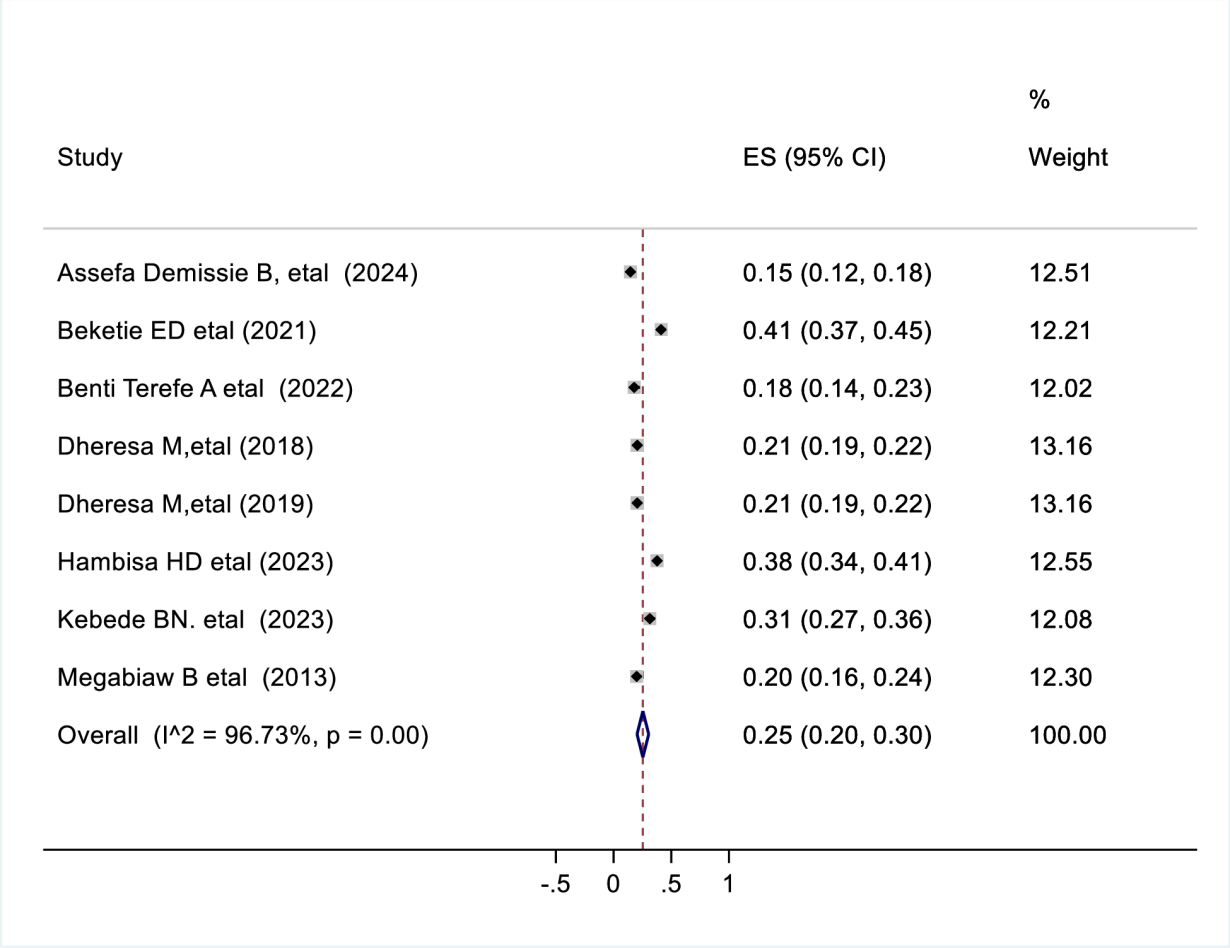
Forest plot shows the pooled prevalence of pelvic floor disorder in Ethiopia, 2024.

The pooled prevalence of pelvic floor disorder from studies in Ethiopia was 25% (95% CI 0.20–0.30). By using a random effect model, a high level of heterogeneity between studies was observed and indicated by the I^2^ statistic (I^2 ^= 96.55%, P = 0.001) (Fig 2).

### Moderator of interest

**Fig 3:**
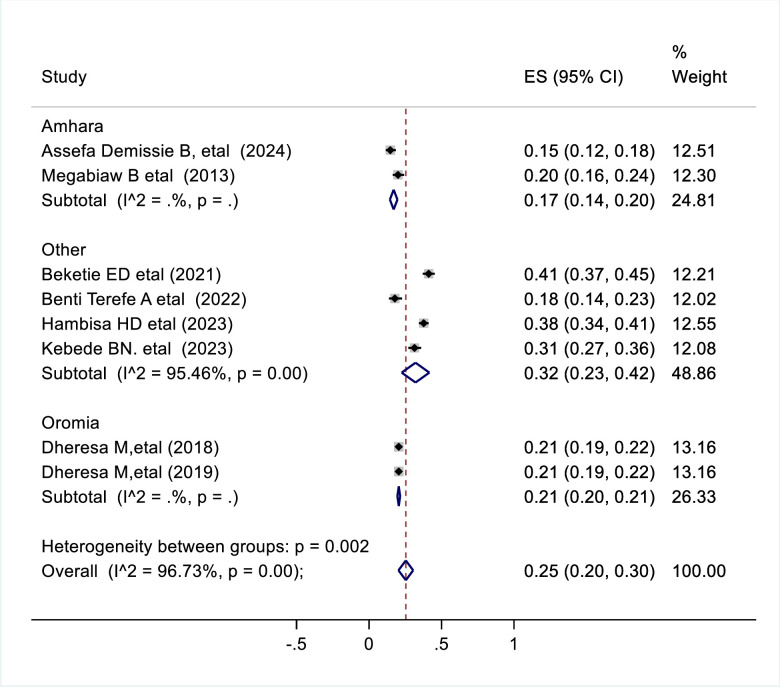
Forest plot shows subgroup analysis result of pooled prevalence of pelvic floor disorder in Ethiopia by region, 2024.

**Fig 4:**
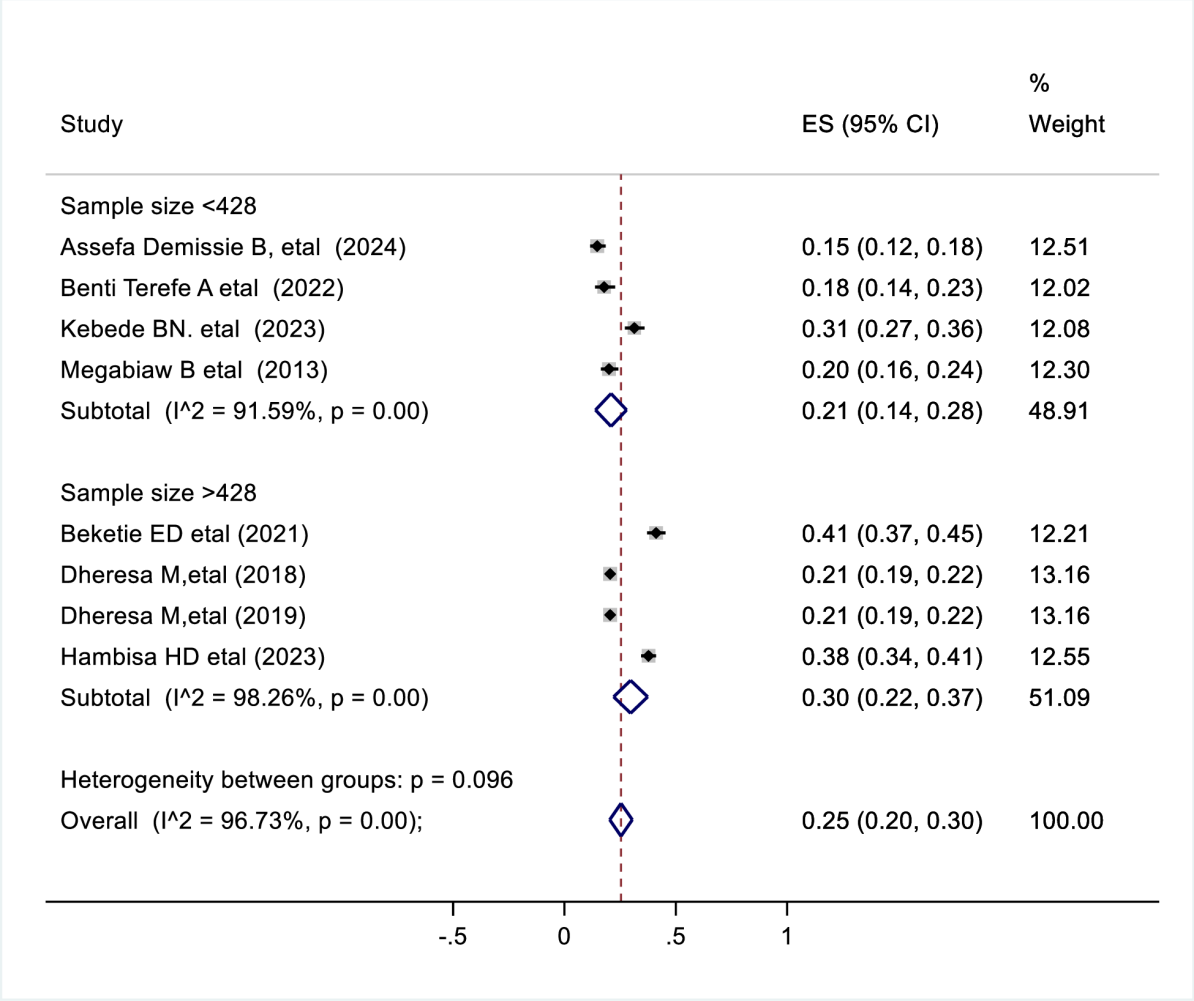
Forest plot shows subgroup analysis result of pooled prevalence of pelvic floor disorder in Ethiopia by sample size, 2024.

Moderators of interest that helped to point out the cause of significant heterogeneity in this review included geographic region of the studies and sample size. According to the constitution of the Federal Democratic Republic of Ethiopia, the geographic area in which the studies took place was categorized as Oromia, Amhara, Southern Nations, Nationalities and Peoples (SNNPR) and Benishangul Gumuz regions. Since the study conducted in Benishangul Gumuz was a single study, it was merged with other nationally conducted studies and labelled as “Other”. To identify possible sources of heterogeneity subgroup analysis was performed using region and sample size. According to our result, the heterogeneity in the prevalence of pelvic floor disorders in the region was significant (p = 0.002) (Fig 3) whereas heterogeneity by sample size was insignificant(p = 0.096) (Fig 4). Based on the results of the subgroup analysis the highest prevalence of pelvic floor disorder is noted from “other” regions (SNNPR and Benishangul Gumuz) which was 32% (95%CI 0.23–0.42) (Fig 3).

### Meta-regression and sensitivity analysis

**Fig 5:**
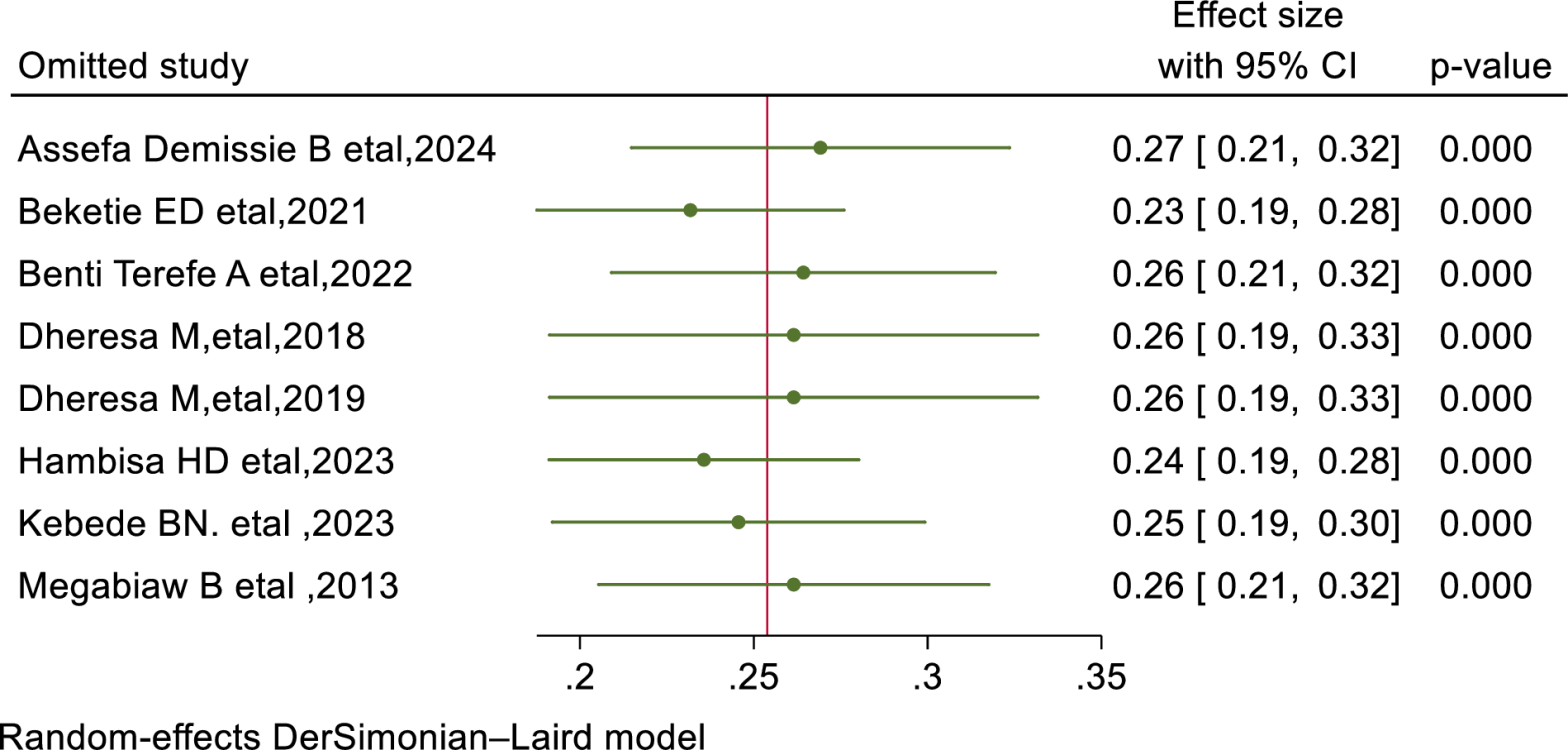
Result of sensitivity analysis of included studies to assess pooled prevalence of pelvic disorder among women in Ethiopia, 2024.

Meta-regression analysis was performed to elucidate the underlying factors contributing to heterogeneity. Based on our systematic review and meta-analysis, the sample size (p = 0.8) was not significant factor for source of heterogeneity whereas publication year (p = 0.01) were found to be significant determinants of the source of heterogeneity. Sensitivity analysis was conducted to evaluate the impact of individual studies on the overall estimate, yet no single study had a significant influence on the pooled estimate of pelvic floor disorders among women in Ethiopia (Fig 5).

### Types of pelvic floor disorder

**Fig 6:**
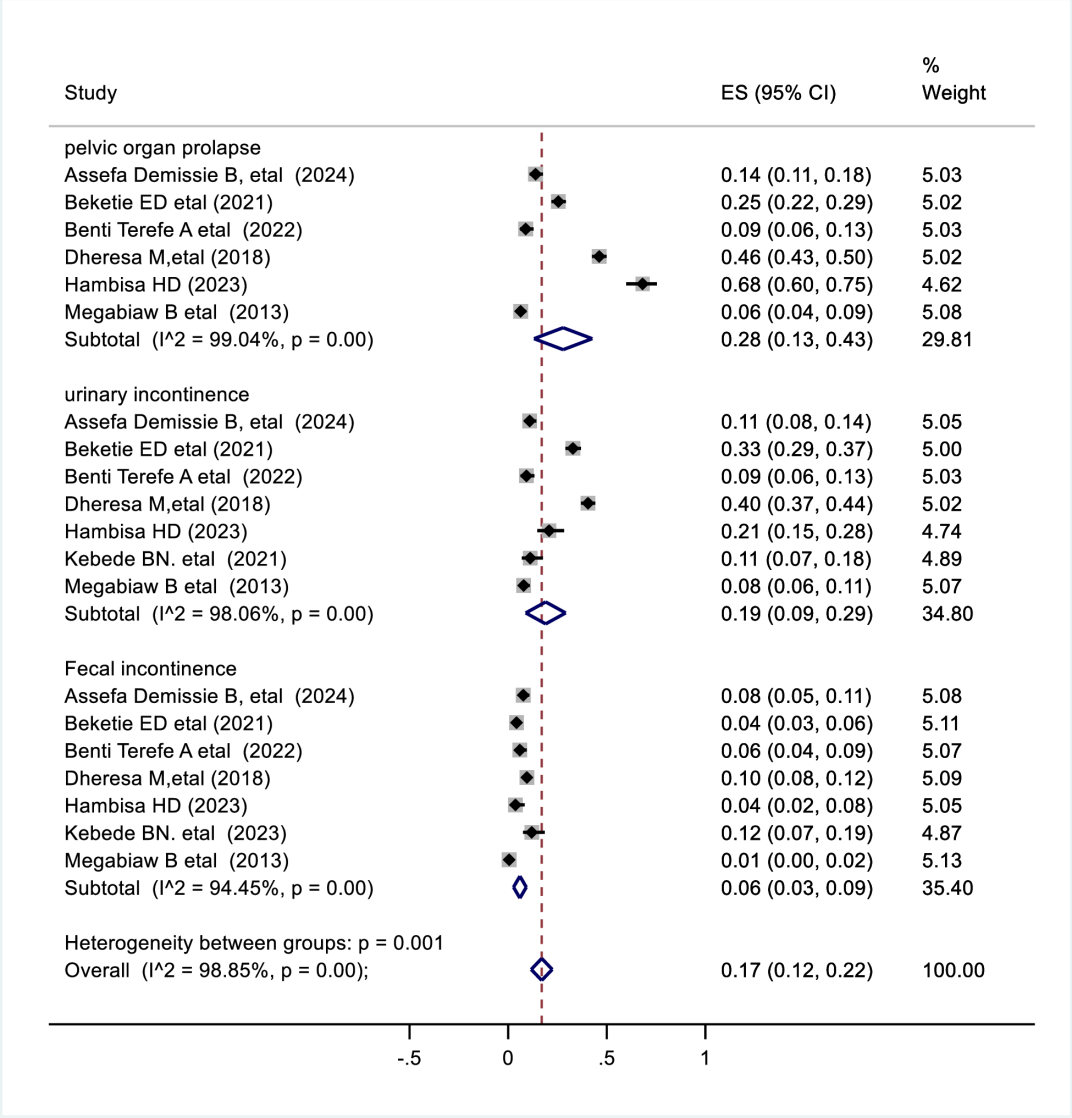
Forest plot shows pooled prevalence of pelvic organ prolapse, urinary incontinence and fecal incontinence among total pelvic floor disorder in Ethiopia, 2024.

According to our finding the combined prevalence of pelvic organ prolapse was 28% (95% CI 0.13–0.43) (Fig 6) followed by urinary incontinence 19% (95% CI 0.09–0.29) (Fig 6). The pooled prevalence of fecal incontinence was 6% (95% CI 0.03–0.09) (Fig 6).

### Risk factors of pelvic floor disorder in Ethiopia

**Fig 7:**
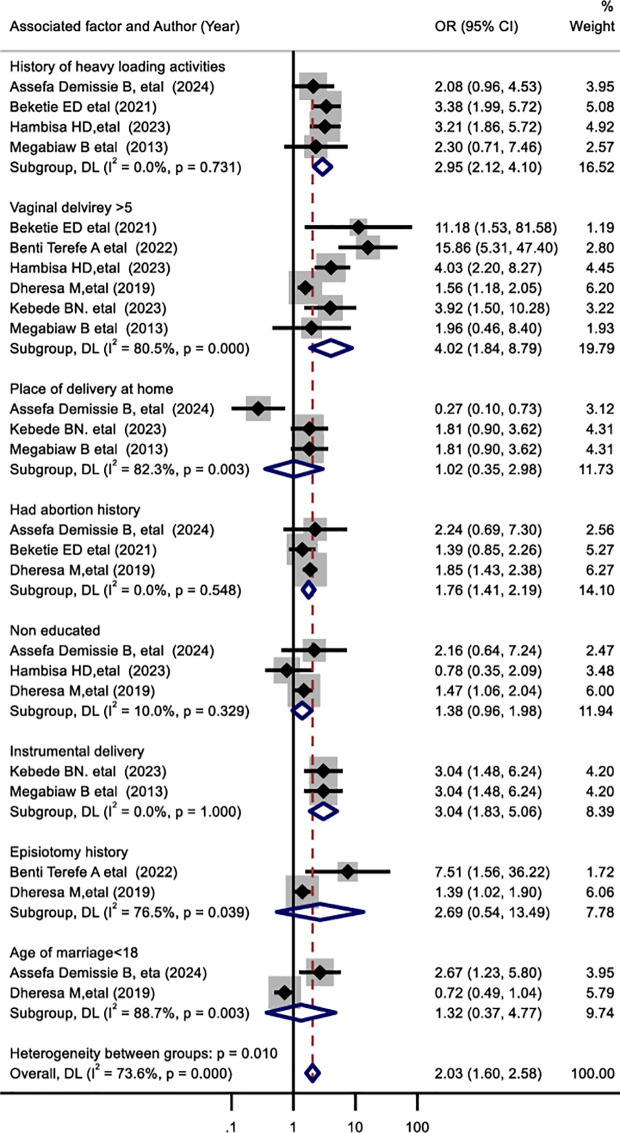
Forest plot shows factors associated with pelvic floor disorders in Ethiopia, 2024.

Seven out of eight primary studies were examined to determine the factors of pelvic floor disorders in Ethiopia. The history of heavy loading activity was examined in four studies [18,19,23,25] (pooled odds ratio: 2.95,95% CI 2.12–4.1). The result indicates that a history of heavy loading activity has no statistically significant association with pelvic floor disorder (Fig 7). Vaginal deliveries of more than or equal to five were examined using data from six studies [19,20,22–25]. The result showed that vaginal delivery of more than or equal to five was significantly associated with pelvic floor disorder in Ethiopia. Pelvic floor disorders were 4.02 times more likely common in mothers who had delivered vaginally more than five times (pooled odds ratio: 4.02,95% CI 1.87–8.79) (Fig 7). The place of delivery at home was assessed in three studies [18,24,25] and showed a statistically significant association with pelvic floor disorder. Pelvic floor disorders were 1.02 times more common in mothers giving birth at home than in mothers giving birth in a health center (pooled odds ratio: 1.02,95% CI 0.35–2.98) (Fig 7). women’s abortion history was assessed from three individual studies [18,19,22] (pooled odds ratio: 1.76,95% CI 1.41–2.19) and its association is not statistically significant. Lack of formal education was examined using data from three studies [18,22,23] and instrumental delivery was also assessed using data from two individual studies [24,25]. Both factors have no statistically significant association with the pelvic floor disorder (Fig 7). The history of episiotomy was examined using data from two studies [20,22]. Pelvic floor disorder was 2.69 times more likely to occur in women with a history of episiotomy than women without a history of episiotomy (pooled odds ratio: 2.69,95% CI 0.54–13.49) (Fig 7). Marriage of marriage under 18 years of age were determined using data from two studies [18,22]. Pelvic floor disorder is 1.32 times more likely to occur in women who married blow age of 18 years old (pooled odds ratio: 1.32,95% CI 0.37–4.77) (Fig 7).

### Publication bias

**Fig 8:**
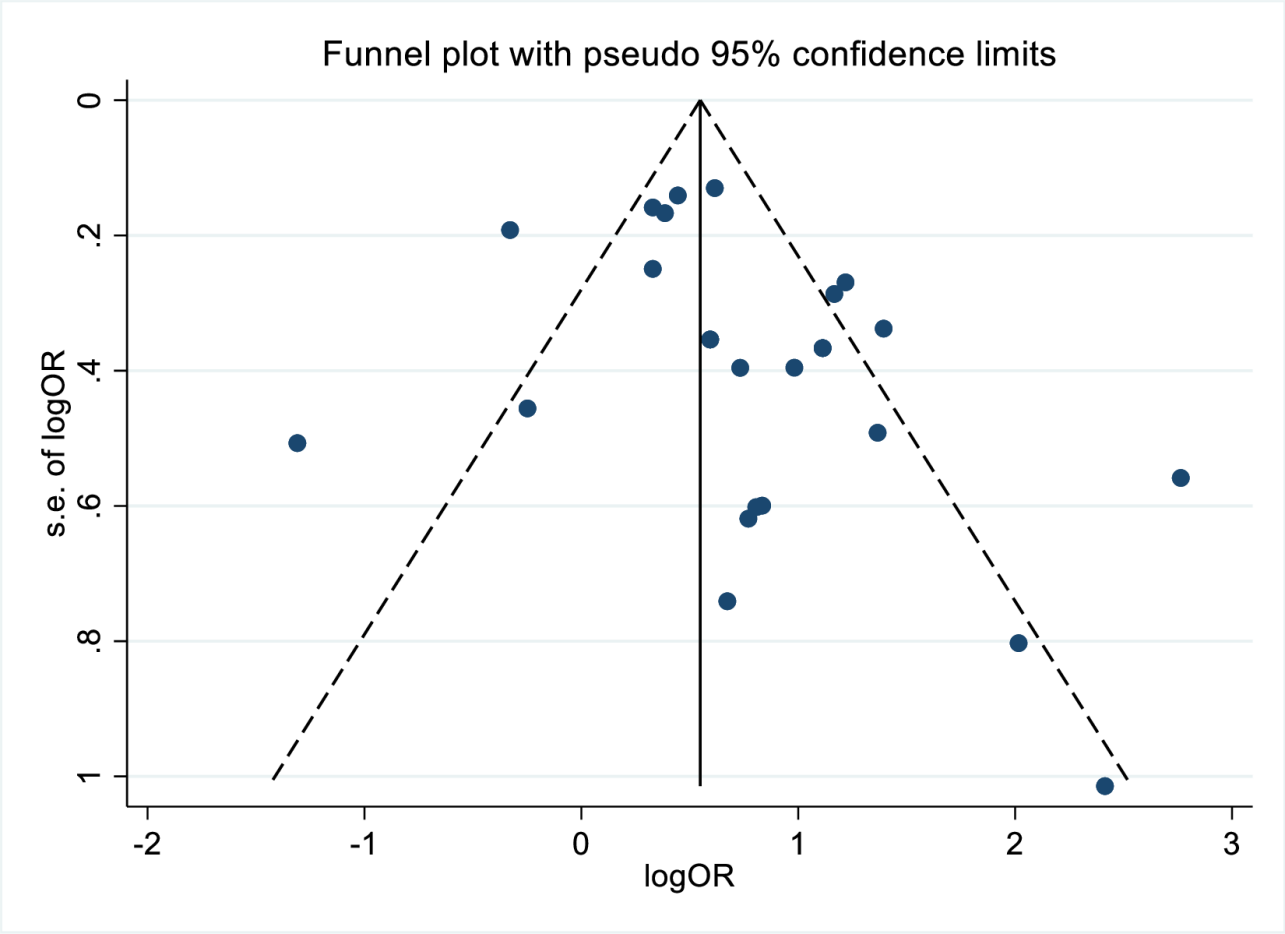
Funnel plot illustrating publication bias of included studies for factors of pelvic floor disorder in Ethiopia, 2024.

The funnel plot shown in Fig 8 provides visual evidence of publication bias associated with the standard error when various factors associated with pelvic floor disorders are considered. The funnel plot showed remarkable symmetry, indicating that the absence of publication bias (Fig 8). The egger test was used to statistically evaluates this bias; the effects of the research on the H0 test revealed non-significant results (Egger test, p = 0.054).

## Discussion

Pelvic floor disorders are one of the unsolved public health problems that affect millions of women worldwide [26]. Pelvic floor disorders have a significant impact on women’s economic, personal, and social dimensions, particularly concerning their daily routines, sexual relationships, mental health, overall quality of life, and the substantial expenses associated with health care [27]. This systematic review and meta-analysis aimed to investigate the pooled prevalence of pelvic floor disorder and its determinate factors among Ethiopian women. This comprehensive systematic review and meta-analysis represents a pioneering effort in Ethiopia as there is currently no documentation detailing the overall prevalence of pelvic floor disorders in the country. The result of this review indicates that the pooled prevalence of pelvic floor disorder in Ethiopia was 25% which is consistent with the pooled prevalence of women in middle and low-income countries(25%) [28] and the US population 25.0% [29]. Pelvic floor disorder in low income countries like Ethiopia is high as compare to developed countries which could be due to limited access to health care,higher fertility rate and higher rate of home delivery [24]. However the pooled prevalence of pelvic floor disorder in this study is lower than in studies in Japan (63.9%) [30], Bangladesh (35.3%) [31] and Turkey(65.3%) [32]. The difference in prevalence between countries might be due to study population, study setting, sociodemographic status and differences in sample size between study participants. According to this systematic review and meta-analysis the pooled prevalence of pelvic organ prolapse was 28% followed by urinary incontinence 19% and fecal incontinence 6% [33]. According to this review pelvic organ prolapse is the most prevalent pelvic floor disorder in Ethiopia. Even if this observation aligns with a systematic review conducted in middle- and low-income countries. Our finding contradicted with finding reported in a review conducted in the Eastern Mediterranean countries, which indicates urinary incontinence as most common pelvic floor disorder with prevalence rates of POP (39%), UI (48%), and FI (39%) [34]. This disparity might stem from the fact that women in Ethiopia tend to undergo more frequent vaginal births and multiple childbirths which lead to pelvic organ prolapse as compared to Eastern Mediterranean nations. Additionally, the rates of urinary incontinence (UI) and fecal incontinence (FI) could differ due to biological or racial factors, with a lower occurrence of urinary incontinence noted among black women in comparison to white women [35]. The lower occurrence of urinary incontinence in back women could be justified due to high urethral closure capacity during maximum pelvic muscle contraction in back women as compare with other race [36].This variation could also be attributed due to differences in sample size, sociocultural factors, health care-seeking behavior, and diagnostic method [37].

The incidence of pelvic floor disorders is influenced by various factors. This systematic review and meta-analysis, it was found that vaginal deliveries exceeding five, home deliveries, a history of episiotomy, and marriages occurring before the age of 18 years had statistically significant correlations with pelvic floor disorders in Ethiopia(P > 0.05). According to this review, mothers who had a vaginal delivery from above five time were 4.02 times more likely to develop pelvic floor disorders. This results in lined with studies conducted in Spain [13],India [38],Tanzania [39] and Australia [40]. This phenomenon may be attributed to the fact that multiparity is one of the significant determinants of levator ani muscle avulsion, while excessive distension, muscle insufficiency, and enlarged hiatal area are critical risk factors for the emergence of pelvic floor disorders [41]. Our result showed that pelvic floor disorders were 1.02 times more common in mothers who gave birth at home than in mothers who gave birth in a health center. This finding is supported by another review [42] and a population-based study [18]. This could be attributed to the reality that home delivery occurs via the vagina, necessitating that the levator muscle and birth canal tissues to expand to more than three times their original length; it is this excessive stretching that leads to muscle tearing e [43]. This review additionally indicated that pelvic floor disorders are 2.69 times more common in women with a history of episiotomy. This is in line with a primary study conducted in Chain [44] and Sweden [45]. This could be because both midline and mediolateral episiotomies reduce pelvic floor muscles strength and may contribute to pelvic floor disorders [46]. However, this is contradicted with studies conducted in Maryland [47] and Italy [48] which report no statistically significant association with pelvic floor disorders. Our review also points out pelvic floor disorders are 1.66 times more common in women who are married under 18 years of age. This is supported by a study conducted in Pakistan [49]. This could be because girls who marry at an early age may become sexually active at a young age, which can affect the pelvic floor structures, and they may also experience multiple births [50].

## Conclusion

Pelvic floor disorder constitutes a significant unresolved public health issue that impacts millions of women globally. Our comprehensive review suggests that the aggregated prevalence of pelvic floor disorders in Ethiopia is comparable to that observed in studies in middle- and low-income countries and the population of United States, but remains lower than the prevalence rates reported in Eastern Mediterranean countries and the population of Bangladesh. Pelvic organ prolapse is the most common pelvic floor disorder in Ethiopia, followed by urinary incontinence and fecal incontinence. Vaginal deliveries exceeding five, home deliveries, a history of episiotomy, and marriage before age 18 were identified as determinant factors affecting pelvic floor disorder in Ethiopia.

### Strength and limitation

The current systematic review and meta-analysis are notable for being the first study to address the pooled prevalence of pelvic floor disorders in Ethiopia. Various databases were used to search for relevant articles. However, it is important to recognize that this review has its limitations; Notably, all included studies were institution based and limited to articles published in English. Furthermore, the differences in sample size of these studies may influence the overall estimate of pelvic floor disorders.

## Supporting information

S1 Table PRISIMA 2020 cheek list. (PDF)

S2 Table List of all studies found through literature searches and reasons for exclusion. (DOCX)

S1 File Search strategies for different data bases. (DOCX)

## Abbreviations

AOR: adjusted odds ratio
CI: Confidence interval
FI: fecal incontinence
JBI: Joanna Briggs Institute
PFD: Pelvic floor dysfunction
POP: pelvic organ prolapses
PRISMA: Preferred Reporting Items for Systematic Reviews and Meta-Analyses
SNNPR: South Nations and Nationalities People’s Region
OR: Odds ratio
UI: urinary incontinence
