# Supplementary material for: Prevalence and associated factors affecting pelvic floor disorder among women in Ethiopia: A systematic review and meta-analysis
Source: PLoS One. 2025 Jul 18;20(7):e0328184. doi: 10.1371/journal.pone.0328184 (PMC12273982; doi:10.1371/journal.pone.0328184)
Supplement: S2 Table — (DOCX) [file pone.0328184.s002.docx]

S2 Table. List of all studies found through literature searches and reasons for exclusion.

| Study Number | Author and study year | Title | Included /Excluded | Reason for exclusion | Data extractor | Date of extraction | Eligibility | Journal |
| --- | --- | --- | --- | --- | --- | --- | --- | --- |
|  | Hambisa HD etal.2023 | Magnitude of symptomatic pelvic floor dysfunction and associated factors amongst women in Western Ethiopia | **Included** | NA | TB | 2024/07/05-2024/08/16 | Eligible | PubMed |
|  | Beketie ED. etal,2021 | Symptomatic pelvic floor disorders and its associated factors in South-Central Ethiopia | **Included** | NA | TB | 2024/07/05-2024/08/16 | Eligible | PubMed |
|  | Gedefaw G, Demis A,2020 | Burden of pelvic organ prolapse in Ethiopia: a systematic review and meta-analysis | Excluded | Excluded by title and abstract | TB | 2024/07/05-2024/08/16 | Not Eligible | PubMed |
|  | Assefa Demissie B etal,2024 | Prevalence and associated factors of symptomatic pelvic floor disorders among women living in Debre Tabor Town, Northwest Amhara, Ethiopia. | **Included** | NA | TB | 2024/07/05-2024/08/16 | Eligible | PubMed |
|  | Siyoum M etal,2024 | Prevalence and risk factors of pelvic organ prolapse among women in Sidama region, Ethiopia: A community-based survey | Excluded | Excluded by title and abstract | TB | 2024/07/05-2024/08/16 | Not eligible | PubMed |
|  | Dheresa M etal,2019 | Factors associated with pelvic floor disorders in Kersa District, eastern Ethiopia: a community-based study | **Included** | NA | TB | 2024/07/05-2024/08/16 | Eligible | PubMed |
|  | Dheresa M etal,2020 | Women’s health seeking behavior for pelvic floor disorders and its associated factors in eastern Ethiopia | Excluded | Excluded by title and abstract | TB | 2024/07/05-2024/08/16 | Not eligible | PubMed |
|  | Dheresa M etal,2018 | One in five women suffer from pelvic floor disorders in Kersa district Eastern Ethiopia: a community-based study | **Included** | NA | TB | 2024/07/05-2024/08/16 | Eligible | PubMed |
|  | Zeleke BM etal,2016 | Symptomatic pelvic floor disorders in community-dwelling older Australian women | Excluded | Excluded by title and abstract | TB | 2024/07/05-2024/08/16 | Not eligible | PubMed |
|  | Zeleke BM etal,2017 | Hypoactive sexual desire dysfunction in community-dwelling older women | Excluded | Excluded by title and abstract | TB | 2024/07/05-2024/08/16 | Not eligible | PubMed |
|  | Zeleke BM etal ,2013 | Depression among women with obstetric fistula, and pelvic organ prolapse in northwest Ethiopia. | Excluded | Excluded by title and abstract | TB | 2024/07/05-2024/08/16 | Not eligible | PubMed |
|  | Megabiaw B etal,2013 | Pelvic floor disorders among women in Dabat district, northwest Ethiopia: a pilot study | **Included** | NA | TB | 2024/07/05-2024/08/16 | Eligible | PubMed |
|  | Zeleke BM etal, 2017 | Vasomotor symptoms are associated with depressive symptoms in community-dwelling older women | Excluded | Excluded by title and abstract | TB | 2024/07/05-2024/08/16 | Not eligible | PubMed |
|  | Benti Terefe A etal,2022 | Determinants of Pelvic Floor Disorders among Women Visiting the Gynecology Outpatient Department in Wolkite University Specialized Center, Wolkite, Ethiopia | **Included** | NA | TB | 2024/07/05-2024/08/16 | Eligible | PubMed |
|  | Blomquist JL etal,2018 | Association of Delivery Mode With Pelvic Floor Disorders After Childbirth | Excluded | Excluded by title and abstract | TB | 2024/07/05-2024/08/16 | Not eligible | PubMed |
|  | Kenne KA etal,2022 | Prevalence of pelvic floor disorders in adult women being seen in a primary care setting and associated risk factors | Excluded | Excluded by title and abstract | TB | 2024/07/05-2024/08/16 | Not eligible | PubMed |
|  | Malaekah H eta,2022 | Prevalence of pelvic floor dysfunction in women in Riyadh, Kingdom of Saudi Arabia: A cross-sectional study | Excluded | Excluded by title and abstract | TB | 2024/07/05-2024/08/16 | Not eligible | PubMed |
|  | Beketie ED. etal,2021 | Symptomatic pelvic floor disorders and its associated factors in South-Central Ethiopia | Excluded | Duplication | TB | 2024/07/05-2024/08/16 | Eligible | Google Scholar |
|  | Assefa Demissie B etal,2024 | Prevalence and associated factors of symptomatic pelvic floor disorders among women living in Debre Tabor Town, Northwest Amhara, Ethiopia | Excluded | Duplication | TB | 2024/07/05-2024/08/16 | Eligible | Google Scholar |
|  | Dheresa M etal,2019 | Factors associated with pelvic floor disorders in Kersa District, eastern Ethiopia: a community-based study | Excluded | Duplication | TB | 2024/07/05-2024/08/16 | Eligible | Google Scholar |
|  | Benti Terefe A etal,2022 | Determinants of Pelvic Floor Disorders among Women Visiting the Gynecology Outpatient Department in Wolkite University Specialized Center, Wolkite, Ethiopia | Excluded | Duplication | TB | 2024/07/05-2024/08/16 | Eligible | Google Scholar |
|  | Hambisa HD etal.2023 | Magnitude of symptomatic pelvic floor dysfunction and associated factors amongst women in Western Ethiopia | Excluded | Duplication | TB | 2024/07/05-2024/08/16 | Eligible | Google Scholar |
|  | Kebede BN etal,2023 | Prevalence of pelvic floor disorder and associated factors among women in Arba Minch Health and Demographic Surveillance Site, Gamo Zone, Southern Ethiopia, 2021 | **Included** | NA | TB | 2024/07/05-2024/08/16 | Eligible | Google Scholar |
|  | Dheresa M etal,2018 | One in five women suffer from pelvic floor disorders in Kersa district Eastern Ethiopia: a community-based study | Excluded | Duplication | TB | 2024/07/05-2024/08/16 | Eligible | Google Scholar |
|  | Dheresa M etal,2020 | Women’s health seeking behavior for pelvic floor disorders and its associated factors in eastern Ethiopia | Excluded | Excluded by title and abstract | TB | 2024/07/05-2024/08/16 | Not eligible | Google Scholar |
|  | Megabiaw B etal,2013 | Pelvic floor disorders among women in Dabat district, northwest Ethiopia: a pilot study | Excluded | Duplication | TB | 2024/07/05-2024/08/16 | Eligible | Google Scholar |
|  | Addisu D etal, 2023 | The prevalence of pelvic organ prolapse and associated factors in Ethiopia | Excluded | Excluded by title and abstract | TB | 2024/07/05-2024/08/16 | Not eligible | Google Scholar |
|  | Abebe SA etal,2023 | Prevalence and Associated Factors of Depression among Women with Advanced Pelvic Organ Prolapse in Northwest Ethiopia | Excluded | Excluded by title and abstract | TB | 2024/07/05-2024/08/16 | Not eligible | Google Scholar |
|  | Tennfjord MK etal ,2023 | Pelvic Floor Disorders and Pelvic Floor Muscle Exercise: A Survey on Knowledge, Attitude, and Practice among Pregnant Women in Northwest Ethiopia | Excluded | Excluded by title and abstract | TB | 2024/07/05-2024/08/16 | Not eligible | Google Scholar |
|  | Gedefaw G etal 2020 | Burden of pelvic organ prolapse in Ethiopia: a systematic review and meta-analysis | Excluded | Excluded by title and abstract | TB | 2024/07/05-2024/08/16 | Not eligible | Google Scholar |
|  | Muche HA etal 2021 | Prevalence and associated factors of pelvic organ prolapse among women attending gynecologic clinic in referral hospitals of Amhara Regional State, Ethiopia | Excluded | Excluded by title and abstract | TB | 2024/07/05-2024/08/16 | Not eligible | Google Scholar |
|  | Dheresa M etal,2019 | Pelvic floor disorders associated with higher-level sexual dysfunction in the Kersa district, Ethiopia | Excluded | Excluded by result not reported | TB | 2024/07/05-2024/08/16 | Not eligible | Google Scholar |
|  | Dina H etal ,2022 | Magnitude of Symptomatic Pelvic Floor Dysfunction and Associated Factors Amongst Women in Western Ethiopia: A Cross-Sectional Study | Excluded | Duplication | TB | 2024/07/05-2024/08/16 | eligible | Google Scholar |
|  | Abebe SA etal, 2024 | Prevalence and associated factors of depression among women with advanced pelvic organ prolapse in Northwest Ethiopia: cross-sectional study | Excluded | Excluded by title and abstract | TB | 2024/07/05-2024/08/16 | Not eligible | Google Scholar |
|  | Peinado-Molina RA etal 2023 | Pelvic floor dysfunction: prevalence and associated factors | Excluded | Excluded by title and abstract | TB | 2024/07/05-2024/08/16 | Not eligible | Google Scholar |
|  | Borsamo A etal,2023 | Associated factors of pelvic organ prolapse among patients at Public Hospitals of Southern Ethiopia: A case-control study design | Excluded | Excluded by title and abstract | TB | 2024/07/05-2024/08/16 | Not eligibility | Google Scholar |
|  | Siyoum M etal,2024 | Prevalence and risk factors of pelvic organ prolapse among women in Sidama region, Ethiopia: A community-based survey | Excluded | Excluded by title and abstract | TB | 2024/07/05-2024/08/16 | Not eligibility | Google Scholar |
|  | Shewarega ES,etal,2023 | Prevalence of symptomatic pelvic organ prolapse and associated factors in Southern Nations, Nationalities, People's Region referral hospitals, Ethiopia | Excluded | Excluded by title and abstract | TB | 2024/07/05-2024/08/16 | Not eligibility | Google Scholar |
|  | Abdissa K etal,2024 | Prevalence and Associated Factors of Anal Incontinence at Six Weeks after Vaginal Delivery: A Cross-sectional Study at Three Teaching Hospitals in Addis Ababa, Ethiopia | Excluded | Excluded by title and abstract | TB | 2024/07/05-2024/08/16 | Not eligibility | Google Scholar |
|  | Edmealem A etal,2023 | Determinants of pelvic organ prolapse among gynecologic patients, Northeastern Ethiopia | Excluded | Excluded by title and abstract | TB | 2024/07/05-2024/08/16 | Not eligibility | Google Scholar |
|  | Tega A etal,2024 | Quality of life and its associated factors among women with pelvic organ prolapse who attend gynecology clinics Southern Ethiopia 2022 | Excluded | Excluded by title and abstract | TB | 2024/07/05-2024/08/16 | Not eligibility | Google Scholar |
|  | Obsa MS etal, 2022 | Risk factors of pelvic organ prolapse at Asella Teaching and Referral Hospital: Unmatched case control study | Excluded | Excluded by title and abstract | TB | 2024/07/05-2024/08/16 | Not eligibility | Google Scholar |
|  | Ali L etal,2022 | Influencing pelvic floor changes on the quality of women's life during and post pregnancy | Excluded | Excluded by title and abstract | TB | 2024/07/05-2024/08/16 | Not eligibility | Google Scholar |
|  | Merga A etal,2023 | Pelvic Organ Prolapse and its Associated Factors Among Women: A Facility Based Cross-sectional Study | Excluded | Excluded by title and abstract | TB | 2024/07/05-2024/08/16 | Not eligibility | Google Scholar |
|  | Tefera Z etal,2023 | Quality of life and its associated factors among women diagnosed with pelvic organ prolapse in Gynecology outpatient department Southern Nations, Nationalities, and Peoples region public referral hospitals, Ethiopia | Excluded | Excluded by title and abstract | TB | 2024/07/05-2024/08/16 | Not eligibility | Google Scholar |
|  | Nardos R etal,2022 | Characterizing persistent urinary incontinence after successful fistula closure: the Uganda experience | Excluded | Excluded by title and abstract | TB | 2024/07/05-2024/08/16 | Not eligibility | Google Scholar |
|  | Tesemma MG,etal,2024 | Prevalence of urinary incontinence and associated factors, its impact on quality of life among pregnant women attending antenatal care at Asella teaching and referral hospital | Excluded | Excluded by title and abstract | TB | 2024/07/05-2024/08/16 | Not eligibility | Google Scholar |
|  | Berhe A etal ,2020 | Urinary incontinence and associated factors among pregnant women attending antenatal care in public health facilities of Mekelle city, Tigray, Ethiopi | Excluded | Excluded by title and abstract | TB | 2024/07/05-2024/08/16 | Not eligibility | Google Scholar |
|  | Mekuria Z etal,2021 | Prevalence and Associated Factors of Utero-Vaginal Prolapse in AddisAbaba, Ethiopia: A Cross-Sectional Study. | Excluded | Excluded by title and abstract | TB | 2024/07/05-2024/08/16 | Not eligibility | Google Scholar |
|  | Zelelow YB etal, 2022 | Pelvic Organ Prolapse and associated factors among Women in Tigray Region, Northern Ethiopia: A Penalized Logistic Regression | Excluded | Excluded by title and abstract | TB | 2024/07/05-2024/08/16 | Not eligibility | Google Scholar |
|  | Alehegn K etal,2020 | Magnitude and associated factors of utero-vaginal prolapse among  women’s visiting gynecology ward from 2016-2019 at Dilla Referral  Hospital, southern Ethiopia | Excluded | Excluded by title and abstract | TB | 2024/07/05-2024/08/16 | Not eligibility | Google Scholar |
|  | Melkie TB etal ,2022 | Translation, reliability, and validity of Amharic versions of the Pelvic Floor Distress Inventory (PFDI-20) and Pelvic Floor Impact Questionnaire (PFIQ-7) | Excluded | Excluded by title and abstract | TB | 2024/07/05-2024/08/16 | Not eligibility | Google Scholar |
|  | Asresie A etal,2016 | Determinants of pelvic organ prolapse among gynecologic patients in Bahir Dar, north West ethiopia: a case–control stud | Excluded | Excluded by title and abstract | TB | 2024/07/05-2024/08/16 | Not eligibility | Google Scholar |
|  | Mingude AB etal,2022 | Determinants of pelvic organ prolapse in Ethiopia: Systematic review and meta-analysis | Excluded | Excluded by title and abstract | TB | 2024/07/05-2024/08/16 | Not eligibility | Google Scholar |
|  | Abebe D etal,2022 | One in ten ever-married women who visited health facilities for various reasons have pelvic organ prolapse in Harari regional state, Eastern Ethiopia | Excluded | Excluded by title and abstract | TB | 2024/07/05-2024/08/16 | Not eligibility | Google Scholar |
|  | Belayneh T etal ,2020 | Pelvic organ prolapse in Northwest Ethiopia: a population-based study | Excluded | Excluded by title and abstract | TB | 2024/07/05-2024/08/16 | Not eligibility | Google Scholar |
|  | Sirage N etal ,2022 | Determinants of pelvic organ prolapse among gynecologic patients attending public referral hospitals in Amhara region, Ethiopia, 2020: Institution-based unmatched case-control study design | Excluded | Excluded by title and abstract | TB | 2024/07/05-2024/08/16 | Not eligibility | Google Scholar |
|  | Lema Z and Yemane Berhane M,2015 | Determinants of Pelvic Organ Prolapse among Gynecological Cases in Wolaita Sodo University Referral Teaching Hospital, Southern Ethiopia: A Case Control Stud | Excluded | Excluded by title and abstract | TB | 2024/07/05-2024/08/16 | Not eligibility | Google Scholar |
|  | Abebe SA etal,2024 | Prevalence and associated factors of depression among women with advanced pelvic organ prolapse in Northwest Ethiopia: cross-sectional study | Excluded | Excluded by title and abstract | TB | 2024/07/05-2024/08/16 | Not eligibility | Google Scholar |
|  | Sori DA etal,2022 | Prevalence and surgical outcomes of stage 3 and 4 pelvic organs prolapse in Jimma university medical center, south west Ethiopia | Excluded | Excluded by title and abstract | TB | 2024/07/05-2024/08/16 | Not eligibility | Google Scholar |
|  | Aiyegbusi AI etal,2023 | Prevalence of pelvic floor dysfunction and associated risk factors among Nulligravida college students: a cross-sectional study | Excluded | Excluded by title and abstract | TB | 2024/07/05-2024/08/16 | Not eligibility | Google Scholar |
|  | Henok A,2017 | Prevalence and factors associated with pelvic organ prolapse among pedestrian back-loading women in bench Maji Zone | Excluded | Excluded by title and abstract | TB | 2024/07/05-2024/08/16 | Not eligibility | Google Scholar |
|  | Seid NY etal ,2024 | Pelvic organ prolapse and reasons for delay in treatment-seeking among women in Dessie Zuriya Woreda, Northeast Ethiopia, 2022 | Excluded | Excluded by title and abstract | TB | 2024/07/05-2024/08/16 | Not eligibility | Google Scholar |
|  | Alamer A etal,2019 | Prevalence of diastasis recti and associated factors among women attending antenatal and postnatal careatmekelle city health facilities, tigray, ethiopia | Excluded | Excluded by title and abstract | TB | 2024/07/05-2024/08/16 | Not eligibility | Google Scholar |
|  | Zeleke BM etal 2013 | Depression among women with obstetric fistula, and pelvic organ prolapse in northwest Ethiopia | Excluded | Excluded by title and abstract | TB | 2024/07/05-2024/08/16 | Not eligibility | Google Scholar |
|  | Abebe D etal ,2022 | One in ten ever-married women who visited health facilities for various reasons have pelvic organ prolapse in Harari regional state, Eastern Ethiopia | Excluded | Excluded by title and abstract | TB | 2024/07/05-2024/08/16 | Not eligibility | Google Scholar |
|  | Badacho AS etal ,2022 | Uterine prolapse and associated factors among reproductive-age women in south-west Ethiopia: A community-based cross-sectional study | Excluded | Excluded by title and abstract | TB | 2024/07/05-2024/08/16 | Not eligibility | Google Scholar |
|  | Mama ST etal,2022 | Pelvic floor disorders/Obstetric fistula | Excluded | Excluded by title and abstract | TB | 2024/07/05-2024/08/16 | Not eligibility | Google Scholar |
|  | Gjerde JL,2018 | Chronic disease among women in a resource-constrained setting. The case of pelvic organ prolapse in rural Ethiopia | Excluded | Excluded by title and abstract | TB | 2024/07/05-2024/08/16 | Not eligibility | Google Scholar |
|  | Islam RM,etal ,2019 | Prevalence of symptomatic pelvic floor disorders in community-dwelling women in low and middle-income countries: a systematic review and meta-analysis | Excluded | Excluded by title and abstract | TB | 2024/07/05-2024/08/16 | Not eligibility | Google Scholar |
|  | Firdisa G etal ,2022 | Determinants of uterovaginal prolapse in Western Ethiopia | Excluded | Excluded by title and abstract | TB | 2024/07/05-2024/08/16 | Not eligibility | Google Scholar |
|  | Al-Badr A etal,2022 | Prevalence of pelvic floor dysfunction: a Saudi national survey | Excluded | Excluded by title and abstract | TB | 2024/07/05-2024/08/16 | Not eligibility | Google Scholar |
|  | Addis NA etal 2024 | Prevalence and associated factors of maternal birth trauma following vaginal delivery at University of Gondar Comprehensive Specialized Hospital, North-West Ethiopia, 2022 | Excluded | Excluded by title and abstract | TB | 2024/07/05-2024/08/16 | Not eligibility | Google Scholar |
|  | Blystad A etal 2018 | Strengthening validity in studies of pelvic floor disorders through qualitative research: an example from Ethiopia | Excluded | Excluded by title and abstract | TB | 2024/07/05-2024/08/16 | Not eligibility | Google Scholar |
|  | Derso F. 2023 | Treatment-Seeking Experiences of Ethiopian Women With Pelvic Organ Prolapse | Excluded | Excluded by title and abstract | TB | 2024/07/05-2024/08/16 | Not eligibility | Google Scholar |
|  | Fleecs JD etal,2024 | Management of the Unhealed Perineal Wound After Proctectomy | Excluded | Excluded by title and abstract | TB | 2024/07/05-2024/08/16 | Not eligibility | Google Scholar |
|  | Borsamo A etal,2021 | Factors associated with delay in seeking treatment among women with pelvic organ prolapse at selected general and referral hospitals of Southern Ethiopia, 2020 | Excluded | Excluded by title and abstract | TB | 2024/07/05-2024/08/16 | Not eligibility | Google Scholar |
|  | Peinado Molina RA etal,2024 | Sexual health in menopausal women with symptoms of pelvic floor disorders | Excluded | Excluded by title and abstract | TB | 2024/07/05-2024/08/16 | Not eligibility | Google Scholar |
|  | Yohannes Z etal,2019 | Knowledge on risk factors of pelvic organ prolapse and associated factors among mothers attending mch clinic, bansa daye primary hospital, south ethiopia … | Excluded | Excluded by title and abstract | TB | 2024/07/05-2024/08/16 | Not eligibility | Google Scholar |
|  | Carroll L etal,2022 | Pelvic organ prolapse: The lived experience | Excluded | Excluded by title and abstract | TB | 2024/07/05-2024/08/16 | Not eligibility | Google Scholar |
|  | Chen C etal,2024 | Effects of different treatment frequencies of electromagnetic stimulation for urinary incontinence in women: study protocol for a randomized controlled trial | Excluded | Excluded by title and abstract | TB | 2024/07/05-2024/08/16 | Not eligibility | Google Scholar |
|  | Nardos R etal ,2020 | Characteristics of persistent urinary incontinence after successful fistula closure in Ethiopian women | Excluded | Excluded by title and abstract | TB | 2024/07/05-2024/08/16 | Not eligibility | Google Scholar |
|  | Ackah M etal. 2022 | Estimated burden, and associated factors of Urinary Incontinence among Sub-Saharan African women aged 15–100 years: A systematic review and meta-analysis | Excluded | Excluded by title and abstract | TB | 2024/07/05-2024/08/16 | Not eligibility | Google Scholar |
|  | Badacho AS etal ,2020 | Uterine prolapse and associated factors among reproductive age women in Dawro zone, southwest Ethiopia: a community based cross sectional study | Excluded | Excluded by title and abstract | TB | 2024/07/05-2024/08/16 | Not eligibility | Google Scholar |
|  | Memon HU and Handa VL,2013 | Vaginal Childbirth and Pelvic Floor Disorder | Excluded | Excluded by title and abstract | TB | 2024/07/05-2024/08/16 | Not eligibility | Google Scholar |
|  | Shitu AW etal, 2023 | Delay in seeking treatment and associated factors among women with pelvic organ prolapse in Wolaita zone, Southern Ethiopia: Hospital based mixed method stud | Excluded | Excluded by title and abstract | TB | 2024/07/05-2024/08/16 | Not eligibility | Google Scholar |
|  | Fisher-Yosef T etal,2024 | Pelvic Floor Dysfunction among Reproductive-Age Women in Israel: Prevalence and Attitudes—A Cross-Sectional Stud | Excluded | Excluded by title and abstract | TB | 2024/07/05-2024/08/16 | Not eligibility | Google Scholar |
|  | Malaekah H etal,2022 | Prevalence of pelvic floor dysfunction in women in Riyadh, Kingdom of Saudi Arabia: A cross-sectional stud | Excluded | Excluded by title and abstract | TB | 2024/07/05-2024/08/16 | Not eligibility | Google Scholar |
|  | Teshome Y etal,2020 | Prevalence of episiotomy and its associated factors in university of gondar comprehensive specialized referral hospital: a retrospective study from Ethiopia | Excluded | Excluded by title and abstract | TB | 2024/07/05-2024/08/16 | Not eligibility | Google Scholar |
|  | Tugume R etal,2022 | Pelvic organ prolapse and its associated factors among women attending the gynecology outpatient clinic at a tertiary hospital in Southwestern Uganda | Excluded | Excluded by title and abstract | TB | 2024/07/05-2024/08/16 | Not eligibility | Google Scholar |
|  | Jokhio AH etal,2020 | Prevalence of pelvic organ prolapse in women, associated factors and impact on quality of life in rural Pakistan: population-based study | Excluded | Excluded by title and abstract | TB | 2024/07/05-2024/08/16 | Not eligibility | Google Scholar |
|  | Sodagar N etal,2022 | Related risk factors for pelvic floor disorders in postpartum women: A cross-sectional study | Excluded | Excluded by title and abstract | TB | 2024/07/05-2024/08/16 | Not eligibility | Google Scholar |
|  | Knol-de Vries GE,and Blanker MH,2022 | Prevalence of co-existing pelvic floor disorders: A scoping review in males and females | Excluded | Excluded by title and abstract | TB | 2024/07/05-2024/08/16 | Not eligibility | Google Scholar |
|  | Siraneh Y,and Workneh A,2018 | Prevalence and Management Outcome of Patients Underwent Vaginal Hysterectomy in Gynecology Ward of Jimma University Medical Center, Southwest Ethiopia | Excluded | Excluded by title and abstract | TB | 2024/07/05-2024/08/16 | Not eligibility | Google Scholar |
|  | Ejigu N etal,2024 | Delay in seeking treatment and associated factors among women with pelvic organ prolapse in Bale Zone, Southeast Ethiopia: a hospital-based cross-sectional study | Excluded | Excluded by title and abstract | TB | 2024/07/05-2024/08/16 | Not eligibility | Google Scholar |
|  | Masenga GG etal,2018 | Prevalence and risk factors for pelvic organ prolapse in Kilimanjaro, Tanzania: A population based study in Tanzanian rural community | Excluded | Excluded by title and abstract | TB | 2024/07/05-2024/08/16 | Not eligibility | Google Scholar |
|  | Gaddam R etal ,2020 | Prevalence of pelvic floor dysfunction in women attending obstetrics  and gynaecology OPD at PES Institute of Medical Sciences asnd  Research, Kuppam | Excluded | Excluded by title and abstract | TB | 2024/07/05-2024/08/16 | Not eligibility | Google Scholar |
|  | Peinado Molina RA etal,2023 | Pelvic floor dysfunction: prevalence and associated factors | Excluded | Excluded by title and abstract | TB | 2024/07/05-2024/08/16 | Not eligibility | Google Scholar |
|  | Chen L etal ,2020 | Association between sexual intercourse frequency and pelvic floor muscle morphology in pregnant wome | Excluded | Excluded by title and abstract | TB | 2024/07/05-2024/08/16 | Not eligibility | Google Scholar |
|  | Xu P etal,2022 | The effectiveness of eHealth interventions on female pelvic floor dysfunction: a systematic review and meta-analysis | Excluded | Excluded by title and abstract | TB | 2024/07/05-2024/08/16 | Not eligibility | Google Scholar |
|  | Saei Ghare Naz M etal,2020 | Polycystic ovary syndrome and pelvic floor dysfunction: a narrative review | Excluded | Excluded by title and abstract | TB | 2024/07/05-2024/08/16 | Not eligibility | Google Scholar |
|  | Brito LG etal,2022 | Age and/or postmenopausal status as risk factors for pelvic organ prolapse development: systematic review with meta-analysis | Excluded | Excluded by title and abstract | TB | 2024/07/05-2024/08/16 | Not eligibility | Google Scholar |
|  | Hakimi S etal 2020 | Prevalence and risk factors of urinary/anal incontinence and pelvic organ prolapse in healthy middle-aged Iranian women | Excluded | Excluded by title and abstract | TB | 2024/07/05-2024/08/16 | Not eligibility | Google Scholar |
|  | Mashayekh-Amiri S etal, 2023 | Psychometric evaluation and cross-cultural adaptation of the Australian Pelvic Floor Questionnaire (APFQ-IR) in Iranian reproductive age women | Excluded | Excluded by title and abstract | TB | 2024/07/05-2024/08/16 | Not eligibility | Google Scholar |
|  | Ai F etal,2018 | Effect of generalized anxiety disorders on the success of pessary treatment for pelvic organ prolapse | Excluded | Excluded by title and abstract | TB | 2024/07/05-2024/08/16 | Not eligibility | Google Scholar |
|  | Goba GK etal, 2019 | Reliability and validity of the Tigrigna version of the Pelvic Floor Distress Inventory–Short Form 20 (PFDI-20) and Pelvic Floor Impact Questionnaire-7 (PFIQ-7) | Excluded | Excluded by title and abstract | TB | 2024/07/05-2024/08/16 | Not eligibility | Google Scholar |
|  | Chi X etal,2023 | The association between the interdelivery interval and early postpartum urinary incontinence in women who had consecutive vaginal deliveries: a retrospective cohort study | Excluded | Excluded by title and abstract | TB | 2024/07/05-2024/08/16 | Not eligibility | Google Scholar |
|  | Addisu D andFentahun B,2024 | Risk factors associated with severe perineal tear at Public Hospitals in Bahir Dar town, Northwest Ethiopi | Excluded | Excluded by title and abstract | TB | 2024/07/05-2024/08/16 | Not eligibility | Google Scholar |
|  | Rashidi F, and Mirghafourvand M, 2023 | Pelvic floor disorder and relevant factors in Iranian women of reproductive age: a cross-sectional study | Excluded | Excluded by title and abstract | TB | 2024/07/05-2024/08/16 | Not eligibility | Google Scholar |
|  | TORGBENU E etal ,2023 | Prevalence and risk factors of urinary incontinence among women in Africa: a systematic review and meta-analysis | Excluded | Excluded by title and abstract | TB | 2024/07/05-2024/08/16 | Not eligibility | Google Scholar |
|  | Rodríguez-Longobardo C etal 2023 | Pelvic Floor Muscle Training Interventions in Female Athletes: A Systematic Review and Meta-analysis | Excluded | Excluded by title and abstract | TB | 2024/07/05-2024/08/16 | Not eligibility | Google Scholar |
|  | Derrar SL etal ,2022 | Knowledge, Attitude, and Practice of Pregnant Women in Jazan, Saudi Arabia Concerning Pelvic Floor Muscle Exercises | Excluded | Excluded by title and abstract | TB | 2024/07/05-2024/08/16 | Not eligibility | Google Scholar |
|  | Neels H etal,2017 | Does pelvic floor muscle contraction early after delivery cause perineal pain in postpartum women? | Excluded | Excluded by title and abstract | TB | 2024/07/05-2024/08/16 | Not eligibility | Google Scholar |
|  | Batista PA etal,2024 | Low back pain, pelvic pain, and associated factors in type 1 diabetic pregnant women | Excluded | Excluded by title and abstract | TB | 2024/07/05-2024/08/16 | Not eligibility | Google Scholar |
|  | Bekele A etal,2016 | Urinary incontinence among pregnant women, following antenatal care at University of Gondar Hospital, North West Ethiopia | Excluded | Excluded by title and abstract | TB | 2024/07/05-2024/08/16 | Not eligibility | Google Scholar |
|  | Tilahun A etal,2024 | Prevalence and Associated Factors of Hemorrhoids and Other Perianal Complications During the Puerperium Among Mothers Who Gave Birth at Debre Tabor Referral Hospital,  Debre Tabor, Ethiopia, 2022 | Excluded | Excluded by title and abstract | TB | 2024/07/05-2024/08/16 | Not eligibility | Google Scholar |
|  | Ilunga-Mbaya E etal,2024 | Exploring risk factors of pelvic organ prolapse at eastern of Democratic Republic of Congo: a case-control study | Excluded | Excluded by title and abstract | TB | 2024/07/05-2024/08/16 | Not eligibility | Google Scholar |
|  | Bø K etal ,2022 | International urogynecology consultation chapter 3 committee 2; conservative treatment of patient with pelvic organ prolapse: Pelvic floor muscle training | Excluded | Excluded by title and abstract | TB | 2024/07/05-2024/08/16 | Not eligibility | Google Scholar |
|  | Birge Ö etal,2021 | Female genital mutilation/cutting in sudan and subsequent pelvic floor dysfunction | Excluded | Excluded by title and abstract | TB | 2024/07/05-2024/08/16 | Not eligibility | Google Scholar |
|  | Peinado Molina RA | Influence of pelvic floor disorders on quality of life in women | Excluded | Excluded by title and abstract | TB | 2024/07/05-2024/08/16 | Not eligibility | Google Scholar |
|  | Krause HG etal,2017 | Mental health screening in women with severe pelvic organ prolapse, chronic fourth-degree obstetric tear and genital tract fistula in western Uganda | Excluded | Excluded by title and abstract | TB | 2024/07/05-2024/08/16 | Not eligibility | Google Scholar |
|  | Pizarro‐Berdichevsky J etal,2016 | Association between pelvic floor disorder symptoms and QoL scores with depressive symptoms among pelvic organ prolapse patients | Excluded | Excluded by title and abstract | TB | 2024/07/05-2024/08/16 | Not eligibility | Google Scholar |
|  | Serin AN, and Birge Ö 2023 | A serious consequence of pelvic organ prolapse: Hydroureteronephrosis | Excluded | Excluded by title and abstract | TB | 2024/07/05-2024/08/16 | Not eligibility | Google Scholar |
|  | Peinado Molina RA etal,2024 | Influence of Pelvic Floor Disorders on Sleep Quality in Women | Excluded | Excluded by title and abstract | TB | 2024/07/05-2024/08/16 | Not eligibility | Google Scholar |
|  | Hammad FT etal,2018 | The degree of bother and healthcare seeking behaviour in women with symptoms of pelvic organ prolapse from a developing gulf country | Excluded | Excluded by title and abstract | TB | 2024/07/05-2024/08/16 | Not eligibility | Google Scholar |
|  | Mashayekh-Amiri S etal,2023 | Translation and measurement properties of the pelvic floor distress inventory-short form (PFDI-20) in Iranian reproductive age women | Excluded | Excluded by title and abstract | TB | 2024/07/05-2024/08/16 | Not eligibility | Google Scholar |
|  | Wang B etal, 2022 | Effect of Hf-rTMS on rehabilitation of chronic pelvic pain syndrome under different intensities : study protocol for a randomized controlled trial. | Excluded | Excluded by title and abstract | TB | 2024/07/05-2024/08/16 | Not eligibility | Google Scholar |
|  | Veit-Rubin N etal 2016 | Association between joint hypermobility and pelvic organ prolapse in women: a systematic review and meta-analysis | Excluded | Excluded by title and abstract | TB | 2024/07/05-2024/08/16 | Not eligibility | Google Scholar |
|  | Gjerde JL etal,2017 | Living with pelvic organ prolapse: voices of women from Amhara region, Ethiopia | Excluded | Excluded by title and abstract | TB | 2024/07/05-2024/08/16 | Not eligibility | Google Scholar |
|  | Roldan CJ etal,2022 | Non-invasive pelvic floor rehabilitation in cancer population: an incomplete cohort | Excluded | Excluded by title and abstract | TB | 2024/07/05-2024/08/16 | Not eligibility | Google Scholar |
|  | Yang F and Liao H,2022 | The Influence of Obstetric Factors on the Occurrence of Pelvic Floor Dysfunction in Women in the Early Postpartum Period | Excluded | Excluded by title and abstract | TB | 2024/07/05-2024/08/16 | Not eligibility | Google Scholar |
|  | Brown HW etal,2022 | International urogynecology consultation chapter 1 committee 2: Epidemiology of pelvic organ prolapse: prevalence, incidence, natural history, and service need | Excluded | Excluded by title and abstract | TB | 2024/07/05-2024/08/16 | Not eligibility | Google Scholar |
|  | Chen L etal,2020 | Performance of self-reported and unsupervised antenatal pelvic floor muscle training and its effects on postpartum stress urinary incontinence among Chinese women: a cohort study | Excluded | Excluded by title and abstract | TB | 2024/07/05-2024/08/16 | Not eligibility | Google Scholar |
|  | Badalian SS etal,2020 | The prevalence of pelvic floor disorders and degree of bother among women attending primary care clinics in Armenia | Excluded | Excluded by title and abstract | TB | 2024/07/05-2024/08/16 | Not eligibility | Google Scholar |
|  | Chen L etal, 2024 | Advances in the Study of Risk Prediction Models for Postpartum Pelvic Floor Dysfunction Diseases | Excluded | Excluded by title and abstract | TB | 2024/07/05-2024/08/16 | Not eligibility | Google Scholar |
|  | Karaaslan Y etal,2022 | Pelvic floor dysfunction symptoms and knowledge level in obese women | Excluded | Excluded by title and abstract | TB | 2024/07/05-2024/08/16 | Not eligibility | Google Scholar |
|  | Saudi RA, and Tosson EE etal,2022 | Prevalence and the degree of distress of pelvic floor disorders symptoms in women seeking primary health care at Ismailia governorate | Excluded | Excluded by title and abstract | TB | 2024/07/05-2024/08/16 | Not eligibility | Google Scholar |
|  | Bekele H etal, 2022 | Magnitude of episiotomy practice and associated factors among women who gave birth at Hiwot Fana Specialized University Hospital, Eastern Ethiopia | Excluded | Excluded by title and abstract | TB | 2024/07/05-2024/08/16 | Not eligibility | Google Scholar |
|  | Schulten SF etal,2022 | Risk factors for pelvic organ prolapse recurrence after sacrospinous hysteropexy or vaginal hysterectomy with uterosacral ligament suspension | Excluded | Excluded by title and abstract | TB | 2024/07/05-2024/08/16 | Not eligibility | Google Scholar |
|  | Gyhagen M.,2013 | Long-term consequences of vaginal delivery on the pelvic floor. A comparison with caesarean section in one-para women | Excluded | Excluded by title and abstract | TB | 2024/07/05-2024/08/16 | Not eligibility | Google Scholar |
|  | Eleje GU etal,2014 | Determinants and management outcomes of pelvic organ prolapse in a low resource setting | Excluded | Excluded by title and abstract | TB | 2024/07/05-2024/08/16 | Not eligibility | Google Scholar |
|  | Jorge CH etal,2024 | Pelvic floor muscle training as treatment for female sexual dysfunction: a systematic review and meta-analysis | Excluded | Excluded by title and abstract | TB | 2024/07/05-2024/08/16 | Not eligibility | Google Scholar |
|  | Hadizadeh-Talasaz Z etal,2024 | Worldwide prevalence of pelvic organ prolapse: a systematic review and meta-analysis | Excluded | Excluded by title and abstract | TB | 2024/07/05-2024/08/16 | Not eligibility | Google Scholar |
|  | Molina RA etal,2024 | Health-related quality of life and psychosocial variables in women with colorectal pelvic floor dysfunction: a cross-sectional study | Excluded | Excluded by title and abstract | TB | 2024/07/05-2024/08/16 | Not eligibility | Google Scholar |
|  | Ntakwinja M etal,2021 | Surgical management of pelvic organ prolapse in a high-volume resource-limited setting | Excluded | Excluded by title and abstract | TB | 2024/07/05-2024/08/16 | Not eligibility | Google Scholar |
|  | Ongenda IO etal,2023 | Opinion: pelvic floor disorders: learning from chronicity & chronic care models | Excluded | Excluded by title and abstract | TB | 2024/07/05-2024/08/16 | Not eligibility | Google Scholar |
|  | Obioha KC etal,2015 | Prevalence and predictors of urinary/anal incontinence after vaginal delivery: prospective study of Nigerian women | Excluded | Excluded by title and abstract | TB | 2024/07/05-2024/08/16 | Not eligibility | Google Scholar |
|  | Gashaw M etal, 2020 | Burden of pelvic girdle pain during pregnancy among women attending ante-natal clinic, Ethiopia: a cross-sectional study | Excluded | Excluded by title and abstract | TB | 2024/07/05-2024/08/16 | Not eligibility | Google Scholar |
|  | Masenga GG etal,2019 | Urinary incontinence and its relation to delivery circumstances: A population-based study from rural Kilimanjaro, Tanzania | Excluded | Excluded by title and abstract | TB | 2024/07/05-2024/08/16 | Not eligibility | Google Scholar |
|  | Braga A and Serati M,2023 | New advances in female pelvic floor dysfunction management | Excluded | Excluded by title and abstract | TB | 2024/07/05-2024/08/16 | Not eligibility | Google Scholar |
|  | Demirtaş HÜ and Doğan A 2024 | Pelvic congestion syndrome | Excluded | Excluded by title and abstract | TB | 2024/07/05-2024/08/16 | Not eligibility | Google Scholar |
|  | Gari AM etal,2023 | Prevalence of stress urinary incontinence and risk factors among Saudi females | Excluded | Excluded by title and abstract | TB | 2024/07/05-2024/08/16 | Not eligibility | Google Scholar |
|  | Jokhio AH,and Mcarthur,2019 | Burden of pelvic girdle pain during pregnancy among women attending ante-natal clinic, Ethiopia:a cross-sectional study | Excluded | Excluded by title and abstract | TB | 2024/07/05-2024/08/16 | Not eligibility | Google Scholar |
|  | da Costa Souza H etal,2023 | Prevalence of pelvic floor disorders and the associated quality of life among institutionalized and noninstitutionalized elderly women: A cross-sectional study | Excluded | Excluded by title and abstract | TB | 2024/07/05-2024/08/16 | Not eligibility | Google Scholar |
|  | Omar MG etal,2023 | Prevalence of pelvic floor dysfunction among women with polycystic ovarian syndrome: a case-control study | Excluded | Excluded by title and abstract | TB | 2024/07/05-2024/08/16 | Not eligibility | Google Scholar |
|  | Burkhart R etal,2021 | Pelvic Floor Dysfunction After Childbirth: Occupational Impact and Awareness of Available Treatment | Excluded | Excluded by title and abstract | TB | 2024/07/05-2024/08/16 | Not eligibility | Google Scholar |
|  | Dietz HP etal , 2012 | Female pelvic floor dysfunction—an imaging perspective | Excluded | Excluded by title and abstract | TB | 2024/07/05-2024/08/16 | Not eligibility | Google Scholar |
|  | Biadgilign S etal, 2013 | A population based survey in Ethiopia using questionnaire as proxy to estimate obstetric fistula prevalence: results from demographic and health survey | Excluded | Excluded by title and abstract | TB | 2024/07/05-2024/08/16 | Not eligibility | Google Scholar |
|  | Thangarajah F etal, 2024 | Care needs and self-induced measures of women with postpartum pelvic floor disorder- Results of a social media-based survey of 2930 women | Excluded | Excluded by title and abstract | TB | 2024/07/05-2024/08/16 | Not eligibility | Google Scholar |
|  | Mittal A etal,2024 | Female Pelvic Floor Disorders in Northern India: Uncommon or Underreported? | Excluded | Excluded by title and abstract | TB | 2024/07/05-2024/08/16 | Not eligibility | Google Scholar |
|  | Dayican DK etal,2023 | Exercise Position to Improve Synergy Between the Diaphragm and Pelvic Floor Muscles in Women With Pelvic Floor Dysfunction: A Cross Sectional Study | Excluded | Excluded by title and abstract | TB | 2024/07/05-2024/08/16 | Not eligibility | Google Scholar |
|  | Khajehei M etal,2015 | An update on sexual function and dysfunction in women | Excluded | Excluded by title and abstract | TB | 2024/07/05-2024/08/16 | Not eligibility | Google Scholar |
|  | Zhang Y etal,2022 | The relationship between pelvic floor functions and vaginal microbiota in 6–8 weeks postpartum women | Excluded | Excluded by title and abstract | TB | 2024/07/05-2024/08/16 | Not eligibility | Google Scholar |
|  | Teixeira FH etal,2020 | Polymorphism rs1800255 from COL3A1 gene and the risk for pelvic organ prolapse | Excluded | Excluded by title and abstract | TB | 2024/07/05-2024/08/16 | Not eligibility | Google Scholar |
|  | Iqbal MH etal,2020 | Diastasis recti abdominis and its associated risk factors in postpartum women | Excluded | Excluded by title and abstract | TB | 2024/07/05-2024/08/16 | Not eligibility | Google Scholar |
|  | Sologuren-García G etal,2024 | Epidemiology of Pelvic Floor Dysfunction in the Tacna Region of Peru, 2023 | Excluded | Excluded by title and abstract | TB | 2024/07/05-2024/08/16 | Not eligibility | Google Scholar |
|  | Adane AA etal ,2013 | Depression among women with obstetric fistula, and pelvic organ prolapse in northwest Ethiopia | Excluded | Excluded by title and abstract | TB | 2024/07/05-2024/08/16 | Not eligibility | Google Scholar |
|  | Mitteroecker P etal,2022 | Did population differences in human pelvic form evolve by drift or selection? | Excluded | Excluded by title and abstract | TB | 2024/07/05-2024/08/16 | Not eligibility | Google Scholar |
|  | Kayembe AT etal,2024 | Pelvic organ prolapse: a cross-sectional study during mass campaign in two hospitals in the city of Kananga in the Democratic Republic of Congo | Excluded | Excluded by title and abstract | TB | 2024/07/05-2024/08/16 | Not eligibility | Google Scholar |
|  | Banihashem S etal,2020 | Impact of biofeedback therapy for pelvic floor-related constipation to improve sexual function | Excluded | Excluded by title and abstract | TB | 2024/07/05-2024/08/16 | Not eligibility | Google Scholar |
|  | Borsamo A etal, 2023 | Quality of Life of Women with Pelvic Organ Prolapse and Associated Factors at Public Hospitals of Central Ethiopia, a Multicenter Study | Excluded | Excluded by title and abstract | TB | 2024/07/05-2024/08/16 | Not eligibility | Google Scholar |
|  | Kaufmann RL etal ,2022 | Normal width of the linea alba, prevalence, and risk factors for diastasis recti abdominis in adults, a cross-sectional study | Excluded | Excluded by title and abstract | TB | 2024/07/05-2024/08/16 | Not eligibility | Google Scholar |
|  | Roa L etal,2021 | Knowledge of pelvic floor disorders amongst immigrant women in Canada | Excluded | Excluded by title and abstract | TB | 2024/07/05-2024/08/16 | Not eligibility | Google Scholar |
|  | Hoque SS etal,2024 | Comparing Patient-Reported Outcome Measures for Pain in Women With Pelvic Floor Disorders: A Scoping Review | Excluded | Excluded by title and abstract | TB | 2024/07/05-2024/08/16 | Not eligibility | Google Scholar |
|  | Ilunga-Mbaya E etal,2023 | Pelvic Organs Prolapse in Low-Resources Countries: Epidemiology, Risk Factors, Quality of Life. Narrative Review | Excluded | Excluded by title and abstract | TB | 2024/07/05-2024/08/16 | Not eligibility | Google Scholar |
|  | Anozie Okechukwu B etal,2020 | Knowledge and Associated Factors of Pelvic Organ Prolapse among Women in Ebonyi State, Nigeria | Excluded | Excluded by title and abstract | TB | 2024/07/05-2024/08/16 | Not eligibility | Google Scholar |
|  | Espitia-De La Hoz FJ,2022 | Prevalence and characterization of pelvic organ prolapse in women from Quindío, Colombia. 2016-2019 | Excluded | Excluded by title and abstract | TB | 2024/07/05-2024/08/16 | Not eligibility | Google Scholar |
|  | Kindie W etal,2023 | Quality of life among women with a diagnosis of pelvic organ prolapse at Felege Hiwot Comprehensive Specialized Hospital, Bahir Dar, Northwest Ethiopia: an institutional based cross-sectional study | Excluded | Excluded by title and abstract | TB | 2024/07/05-2024/08/16 | Not eligibility | Google Scholar |
|  | Sweta K etal,2021 | Assessment of the effect of Mulabandha yoga therapy in healthy women, stigmatized for pelvic floor dysfunctions: A randomized controlled trial | Excluded | Excluded by title and abstract | TB | 2024/07/05-2024/08/16 | Not eligibility | Google Scholar |
|  | Alzahrani RE,2024 | Pelvic Floor Complications During Childbirth-a Systematic Review and Meta-Analysis | Excluded | Excluded by title and abstract | TB | 2024/07/05-2024/08/16 | Not eligibility | Google Scholar |
|  | Szatmári É etal,2023 | Hungarian Women’s Health Care Seeking Behavior and Knowledge of Urinary Incontinence and Pelvic Organ Prolapse: A Cross-Sectional Study | Excluded | Excluded by title and abstract | TB | 2024/07/05-2024/08/16 | Not eligibility | Google Scholar |
|  | Gumanga SK etal,2014 | Social demographic characteristics of women with pelvic organ prolapse at the Tamale Teaching Hospital, Ghana | Excluded | Excluded by title and abstract | TB | 2024/07/05-2024/08/16 | Not eligibility | Google Scholar |
|  | Cimsit C etal,2016 | Prevalence of dynamic magnetic resonance imaging-identified pelvic organ prolapse in pre- and postmenopausal women without clinically evident pelvic organ descent | Excluded | Excluded by title and abstract | TB | 2024/07/05-2024/08/16 | Not eligibility | Google Scholar |
|  | Gupta S etal,2023 | Comparative Evaluation of Effectiveness of Pelvic Floor Strengthening and Myokinetic Active Release of Trigger Points in Reducing Dysmenorrhea | Excluded | Excluded by title and abstract | TB | 2024/07/05-2024/08/16 | Not eligibility | Google Scholar |
|  | Ansari MK etal, 2022 | Pelvic organ prolapse in perimenopausal and menopausal women | Excluded | Excluded by title and abstract | TB | 2024/07/05-2024/08/16 | Not eligibility | Google Scholar |
|  | Larouche M etal,2020 | Depression, Anxiety, and Pelvic Floor Symptoms Before  and After Surgery for Pelvic Floor Dysfunction | Excluded | Excluded by title and abstract | TB | 2024/07/05-2024/08/16 | Not eligibility | Google Scholar |
|  | Levy G etal,2020 | Overactive Bladder Syndrome Treatments and Their Effect on Female Sexual Function: A Review | Excluded | Excluded by title and abstract | TB | 2024/07/05-2024/08/16 | Not eligibility | Google Scholar |
|  | Kenne K etal, 2020 | Surgical Management of Pelvic Organ Prolapse in Ethiopian Women: What Is the Preferred Approach? | Excluded | Excluded by title and abstract | TB | 2024/07/05-2024/08/16 | Not eligibility | Google Scholar |
|  | Ai FF etal,2018 | Effect of generalized anxiety disorders on the success of pessary treatment for pelvic organ prolapse | Excluded | Excluded by title and abstract | TB | 2024/07/05-2024/08/16 | Not eligibility | Google Scholar |
|  | Singh R etal,2024 | Postpartum Pelvic floor disorder in Primiparous women - A prospective observational study | Excluded | Excluded by title and abstract | TB | 2024/07/05-2024/08/16 | Not eligibility | Google Scholar |
|  | Devkota HR etal,2020 | Bio-mechanical risk factors for uterine prolapse among women living in the hills of west Nepal: A case-control study | Excluded | Excluded by title and abstract | TB | 2024/07/05-2024/08/16 | Not eligibility | Google Scholar |
|  | Dos Santos GB etal,2023 | Pelvic floor dysfunctions in women with fibromyalgia: A cross-sectional study | Excluded | Excluded by title and abstract | TB | 2024/07/05-2024/08/16 | Not eligibility | Google Scholar |
|  | Abrar S etal,2023 | Female Urinary Incontinence: Frequency, Risk Factors, and Impact on the Quality of Life of Pregnant Pakistani Women | Excluded | Excluded by title and abstract | TB | 2024/07/05-2024/08/16 | Not eligibility | Google Scholar |
|  | UK NG ,2021 | Management of pelvic girdle pain in pregnancy: Antenatal care | Excluded | Excluded by title and abstract | TB | 2024/07/05-2024/08/16 | Not eligibility | Google Scholar |
|  | Maroyi R etal,2021 | Prevalence of urinary incontinence in pregnant and postpartum women in the Democratic Republic of Congo | Excluded | Excluded by title and abstract | TB | 2024/07/05-2024/08/16 | Not eligibility | Google Scholar |
|  | Nzinga Luzolo AM etal,2024 | Epidemiological Profile and Attitudes of Pregnant Women Toward Urinary Incontinence: A Single-Center Cross-Sectional Study | Excluded | Excluded by title and abstract | TB | 2024/07/05-2024/08/16 | Not eligibility | Google Scholar |
|  | Kone AM etal,2024 | Non-Invasive Pelvic Floor Rehabilitation in Cancer Population | Excluded | Excluded by title and abstract | TB | 2024/07/05-2024/08/16 | Not eligibility | Google Scholar |
|  | Kassa DW etal, 2019 | Validation of the Pelvic Organ Prolapse Simple Screening Inventory (POPSSI) in a population of Ethiopian women | Excluded | Excluded by title and abstract | TB | 2024/07/05-2024/08/16 | Not eligibility | Google Scholar |
|  | Ayele TA, etal,2016 | Prevalence and Associated Factors of Antenatal Depression among Women Attending Antenatal Care Service at Gondar University Hospital, Northwest Ethiopia | Excluded | Excluded by title and abstract | TB | 2024/07/05-2024/08/16 | Not eligibility | Google Scholar |
|  | Geidam AD etal,2022 | Prevalence and Risk Factors Associated with the Development of Severe Pelvic Organ Prolapse in the University of Maiduguri Teaching Hospital, Nigeria | Excluded | Excluded by title and abstract | TB | 2024/07/05-2024/08/16 | Not eligibility | Google Scholar |
|  | Okeahialam NA etal,2021 | Anal and urinary incontinence in nulliparous women – Prevalence and associated risk factors | Excluded | Excluded by title and abstract | TB | 2024/07/05-2024/08/16 | Not eligibility | Google Scholar |
|  | Ghanbari Z etal,2020 | Rate of urinary tract infection after urodynamic study in pelvic floor clinic | Excluded | Excluded by title and abstract | TB | 2024/07/05-2024/08/16 | Not eligibility | Google Scholar |
|  | Siyoum M etal,2023 | Reliability and validity of the Sidaamu Afoo version of the pelvic organ prolapse symptom score questionnaire | Excluded | Excluded by title and abstract | TB | 2024/07/05-2024/08/16 | Not eligibility | Google Scholar |
|  | Abu Raddaha AH, and Nasr EH,2022 | Kegel Exercise Training Program among Women with Urinary Incontinence | Excluded | Excluded by title and abstract | TB | 2024/07/05-2024/08/16 | Not eligibility | Google Scholar |
|  | Wang J etal,2024 | A visualization analysis of hotspots and global trends on pelvic floor dysfunction in cervical cancer | Excluded | Excluded by title and abstract | TB | 2024/07/05-2024/08/16 | Not eligibility | Google Scholar |
|  | Parlas M,and Bilgic D,2024 | Awareness of urinary incontinence in pregnant women as a neglected issue: a cross-sectional study | Excluded | Excluded by title and abstract | TB | 2024/07/05-2024/08/16 | Not eligibility | Google Scholar |
|  | Mengistu Z etal,2021 | Is pelvic floor muscle contractility an important factor in anal incontinence? | Excluded | Excluded by title and abstract | TB | 2024/07/05-2024/08/16 | Not eligibility | Google Scholar |
|  | Khalmanova K,2020 | Nurses’ and midwives’ knowledge of Female Pelvic Floor Disorders in Kazakhstan | Excluded | Excluded by title and abstract | TB | 2024/07/05-2024/08/16 | Not eligibility | Google Scholar |
|  | Oppong EE etal,2021 | Spectrum of anxiety and depression reported in reproductive-aged women diagnosed with gynaecological disorders at a tertiary healthcare facility in Ghana | Excluded | Excluded by title and abstract | TB | 2024/07/05-2024/08/16 | Not eligibility | Google Scholar |
|  | SHETTY R etal,2024 | Assessment of the knowledge of urinary incontinence and pelvic organ prolapse in postpartum women - A cross sectional study | Excluded | Excluded by title and abstract | TB | 2024/07/05-2024/08/16 | Not eligibility | Google Scholar |
|  | Abedian S,and Iravani M etal,2020 | childbirth and the pelvic floor muscles and strategies to prevent the disorder: a review article | Excluded | Excluded by title and abstract | TB | 2024/07/05-2024/08/16 | Not eligibility | Google Scholar |
|  | Prateek S etal, 2021 | Women's experiences and perception of symptomatic pelvis organ prolapse: A Cross sectional study from Uttarakhand, India | Excluded | Excluded by title and abstract | TB | 2024/07/05-2024/08/16 | Not eligibility | Google Scholar |
|  | Ismail A, and Bibi I,2024 | Unveiling Pelvic Floor Health: Understanding Awareness,Perspectives and Habits in Pakistani Women of Reproductive Age: Unveiling Pelvic Floor Health | Excluded | Excluded by title and abstract | TB | 2024/07/05-2024/08/16 | Not eligibility | Google Scholar |
|  | Anyaka C etal,2022 | Pelvic organ prolapse (POP) managed at Jos University Teaching Hospital, Jos Nigeria (JUTH): a 10-year review | Excluded | Excluded by title and abstract | TB | 2024/07/05-2024/08/16 | Not eligibility | Google Scholar |
|  | de Vasconcelos VS etal,2021 | Frequency and Factors Associated with Urinary Incontinence in Pregnant Adolescents: A Cross-Sectional Study | Excluded | Excluded by title and abstract | TB | 2024/07/05-2024/08/16 | Not eligibility | Google Scholar |
|  | Danilina OA, and Volkov VG,2022 | Risk factors for pelvic prolapse in women of reproductive age | Excluded | Excluded by title and abstract | TB | 2024/07/05-2024/08/16 | Not eligibility | Google Scholar |
|  | Soliman HF etal, 2023 | Effect of Mayan Abdominal Massage Technique on Sexuality and Quality of Life among Women with Uterine Prolapse | Excluded | Excluded by title and abstract | TB | 2024/07/05-2024/08/16 | Not eligibility | Google Scholar |
|  | Gjerde JL etal, 2018 | Life after pelvic organ prolapse surgery: a qualitative study in Amhara region, Ethiopia | Excluded | Excluded by title and abstract | TB | 2024/07/05-2024/08/16 | Not eligibility | Google Scholar |
|  | Rajole K etal, 2022 | Effect of stretching exercises on menstrual pain among adolescent girls | Excluded | Excluded by title and abstract | TB | 2024/07/05-2024/08/16 | Not eligibility | Google Scholar |
|  | Haque F, and Sarker S,2022 | Incidence and Study of Sociodemographic Factors & Other Risk Factor fors Pelvic Organ Prolapse | Excluded | Excluded by title and abstract | TB | 2024/07/05-2024/08/16 | Not eligibility | Google Scholar |
|  | Anumba DO etal,2020 | Postnatal pelvic floor muscle stiffness measured by vaginal elastometry in women with obstetric anal sphincter injury: a pilot study | Excluded | Excluded by title and abstract | TB | 2024/07/05-2024/08/16 | Not eligibility | Google Scholar |
|  | Fitz FF etal,2023 | PEOPLE: Lifestyle and comorbidities as risk factors for pelvic organ prolapse—a systematic review and meta-analysis PEOPLE: PElvic Organ Prolapse Lifestyle comorbidities | Excluded | Excluded by title and abstract | TB | 2024/07/05-2024/08/16 | Not eligibility | Google Scholar |
|  | Byamugisha J etal,2023 | Characteristics and outcomes of patients with pelvic organ prolapse: an analysis of data from Mulago National Referral Hospital from 2007–2016 | Excluded | Excluded by title and abstract | TB | 2024/07/05-2024/08/16 | Not eligibility | Google Scholar |
|  | Gurjar B, and Kedar K,2017 | Pelvic organ prolapse- a concern | Excluded | Excluded by title and abstract | TB | 2024/07/05-2024/08/16 | Not eligibility | Google Scholar |
|  | Canday M, and Yurtkal A 2023 | Evaluation and perspectives on hysterosalpingography (HSG) procedure in infertility: a comprehensive stud | Excluded | Excluded by title and abstract | TB | 2024/07/05-2024/08/16 | Not eligibility | Google Scholar |
|  | Mahmoud NA etal, 2023 | Women Awareness and Preventive Measures Practice Regarding Pelvic Organs Prolaps | Excluded | Excluded by title and abstract | TB | 2024/07/05-2024/08/16 | Not eligibility | Google Scholar |
|  | Tiwari S etal, 2024 | Study to Evaluate the Relation Between the Risk Factors and Pelvic Organ Prolapse Quantification Stages Res | Excluded | Excluded by title and abstract | TB | 2024/07/05-2024/08/16 | Not eligibility | Google Scholar |
|  | Geidam AD, and Goje JD,2023 | A 10-year review of records of patients managed for pelvic organ prolapse at a University Teaching Hospital, in Northeast Nigeria. | Excluded | Excluded by title and abstract | TB | 2024/07/05-2024/08/16 | Not eligibility | Google Scholar |
|  | McAlarnen LA etal,2021 | Pelvic floor myofascial pain in gynecology oncology patients: A pilot study | Excluded | Excluded by title and abstract | TB | 2024/07/05-2024/08/16 | Not eligibility | Google Scholar |
|  | Upganlawar DS etal, 2023 | Efficacy of Connective Tissue Therapy and Abdominal Stretching Exercises in Individuals With Primary Dysmenorrhea: A Review | Excluded | Excluded by title and abstract | TB | 2024/07/05-2024/08/16 | Not eligibility | Google Scholar |
|  | Malaekah H,etal,2022 | Prevalence of pelvic floor dysfunction in women in Riyadh, Kingdom of Saudi Arabia: a cross-sectional study | Excluded | Excluded by title and abstract | TB | 2024/07/05-2024/08/16 | Not eligibility | Google Scholar |
|  | Demir E etal,2023 | Effects of the Oxytocin Hormone on Pelvic Floor Muscles in Pregnant Rats | Excluded | Excluded by title and abstract | TB | 2024/07/05-2024/08/16 | Not eligibility | Google Scholar |
|  | Jia Y etal,2023 | Risk factors accounting for anal incontinence during the first year after vaginal delivery—A case control study in China | Excluded | Excluded by title and abstract | TB | 2024/07/05-2024/08/16 | Not eligibility | Google Scholar |
|  | Beksac AT etal, 2017 | Gestational urinary incontinence in nulliparous pregnancy-a pilot study | Excluded | Excluded by title and abstract | TB | 2024/07/05-2024/08/16 | Not eligibility | Google Scholar |
|  | Belayneh T etal,2019 | Validation of the Amharic version of the pelvic organ prolapse symptom score (POP-SS) | Excluded | Excluded by title and abstract | TB | 2024/07/05-2024/08/16 | Not eligibility | Google Scholar |
|  | Mao YJ etal,2020 | The relationship between vaginal microenvironment and pelvic dysfunctional diseases in Chinese women | Excluded | Excluded by title and abstract | TB | 2024/07/05-2024/08/16 | Not eligibility | Google Scholar |
|  | Bahiyah Abdullah M etal,2023 | Awareness of pelvic organ prolapse and attitude towards its treatment among Malaysian women | Excluded | Excluded by title and abstract | TB | 2024/07/05-2024/08/16 | Not eligibility | Google Scholar |
|  | Çıtak G, and Demirtürk F etal,2021 | Urinary incontinence during pregnancy and determination of the factors affecting it | Excluded | Excluded by title and abstract | TB | 2024/07/05-2024/08/16 | Not eligibility | Google Scholar |
|  | Mishra S,etal,2021 | Clinical Epidemiology of Uterovaginal Prolapse | Excluded | Excluded by title and abstract | TB | 2024/07/05-2024/08/16 | Not eligibility | Google Scholar |
|  | Cohen KE etal,2020 | Enhancing behavioral treatment for women with pelvic floor disorders | Excluded | Excluded by title and abstract | TB | 2024/07/05-2024/08/16 | Not eligibility | Google Scholar |
|  | Elneil S,2016 | Female sexual dysfunction in female genital mutilation | Excluded | Excluded by title and abstract | TB | 2024/07/05-2024/08/16 | Not eligibility | Google Scholar |
|  | Naidoo TD, and Moodley J,2015 | Obstetric perineal injury: risk factors and prevalence in a resource-constrained setting | Excluded | Excluded by title and abstract | TB | 2024/07/05-2024/08/16 | Not eligibility | Google Scholar |
|  | Thomas DN, 2022 | Improved body image after uterovaginal prolapse surgery with or without hysterectomy | Excluded | Excluded by title and abstract | TB | 2024/07/05-2024/08/16 | Not eligibility | Google Scholar |
|  | Blyholder L etal,2017 | Exercise behaviors and health conditions of runners after childbirth | Excluded | Excluded by title and abstract | TB | 2024/07/05-2024/08/16 | Not eligibility | Google Scholar |
|  | Javed H etal,2022 | Frequency of urinary incontinence among pregnant women: A cross-sectional survey | Excluded | Excluded by title and abstract | TB | 2024/07/05-2024/08/16 | Not eligibility | Google Scholar |
|  | Parvathavarthini K, and Vanusha A etal,2019 | Clinical epidemiological study of uterine prolapse | Excluded | Excluded by title and abstract | TB | 2024/07/05-2024/08/16 | Not eligibility | Google Scholar |
|  | Okui N etal,2023 | Long-term improvement in urinary incontinence in an elite female athlete through the laser treatment | Excluded | Excluded by title and abstract | TB | 2024/07/05-2024/08/16 | Not eligibility | Google Scholar |
|  | Lai YT etal,2022 | Review of school violence in Taiwan: prevalence, types, and strategies | Excluded | Excluded by title and abstract | TB | 2024/07/05-2024/08/16 | Not eligibility | Google Scholar |
|  | Fang-Fang A etal, 2018 | Association of stress-related disorders with subsequent autoimmune disease | Excluded | Excluded by title and abstract | TB | 2024/07/05-2024/08/16 | Not eligibility | Google Scholar |
|  | Dian SY etal,2024 | Management of multiple pelvic organ prolapse due to pulmonary tuberculosis in a suburban hospital | Excluded | Excluded by title and abstract | TB | 2024/07/05-2024/08/16 | Not eligibility | Google Scholar |
|  | Kurniawati EM etal,2023 | Risk factors of stress urinary incontinence in pelvic organ prolapse patients | Excluded | Excluded by title and abstract | TB | 2024/07/05-2024/08/16 | Not eligibility | Google Scholar |
|  | Rastegar R etal,2023 | Comparing the Differences in Children’s Perceptions of Parental Conflict, Self-Differentiation and Being Triangulated in Individuals with and without Functional Constipation Due to Pelvic Floor Dysfunction | Excluded | Excluded by title and abstract | TB | 2024/07/05-2024/08/16 | Not eligibility | Google Scholar |
|  | Kurniawati EM etal, 2021 | Opportunities in Cases of Stress Urinary Incontinence | Excluded | Excluded by title and abstract | TB | 2024/07/05-2024/08/16 | Not eligibility | Google Scholar |
|  | Shanka NY ,2024 | The Impact of Labor Complications on Episiotomy Rates in a Rural Ethiopian Hospital | Excluded | Excluded by title and abstract | TB | 2024/07/05-2024/08/16 | Not eligibility | Google Scholar |
|  | Abd Elgawad MS etal,2022 | Comparison Between the Effect of Electromagnetic Stimulation Chair with or Without Birth Ball on Pelvic Floor Muscle Dysfunction | Excluded | Excluded by title and abstract | TB | 2024/07/05-2024/08/16 | Not eligibility | Google Scholar |
|  | Brown K etal,2024 | Experiences of Indigenous peoples living with pelvic health conditions | Excluded | Excluded by title and abstract | TB | 2024/07/05-2024/08/16 | Not eligibility | Google Scholar |
|  | Filippini M etal,2023 | A Qualitative and Quantitative Study to Evaluate the Effectiveness and Safety of Magnetic Stimulation in Women with Urinary Incontinence Symptoms and Pelvic Floor Disorders | Excluded | Excluded by title and abstract | TB | 2024/07/05-2024/08/16 | Not eligibility | Google Scholar |
|  | Kato E etal,2022 | Pelvic organ prolapse and Japanese lifestyle | Excluded | Excluded by title and abstract | TB | 2024/07/05-2024/08/16 | Not eligibility | Google Scholar |
|  | Hailu M, and Amsalu H,2024 | Impact of Physical Activity During Pregnancy on Delivery | Excluded | Excluded by title and abstract | TB | 2024/07/05-2024/08/16 | Not eligibility | Google Scholar |
|  | Zhou YN etal ,2020 | Study on the effect of electric current intensity stimulation combined with biofeedback pelvic floor muscle training on postpartum pelvic floor dysfunction. | Excluded | Excluded by title and abstract | TB | 2024/07/05-2024/08/16 | Not eligibility | Google Scholar |
|  | Lew SQ and Robinson III JK etal,2014 | A conservative approach to peritoneal dialysis-associated rectocele. | Excluded | Excluded by title and abstract | TB | 2024/07/05-2024/08/16 | Not eligibility | Google Scholar |
|  | Eid Abd El-hamid N etal,2023 | Assessment of Womens Knowledge, Practices and Attitudes regarding Uterine Prolapse | Excluded | Excluded by title and abstract | TB | 2024/07/05-2024/08/16 | Not eligibility | Google Scholar |
|  | Jeppson PC etal,2024 | Impact of Lifestyle Modifications on the Prevention and Treatment of Pelvic Organ Prolapse | Excluded | Excluded by title and abstract | TB | 2024/07/05-2024/08/16 | Not eligibility | Google Scholar |
|  | Roy TB, Das P etal,2024 | Unique contribution of maternal factors and its association with anemia among under 5 children in Indian context | Excluded | Excluded by title and abstract | TB | 2024/07/05-2024/08/16 | Not eligibility | Google Scholar |
|  | Xu C etal,2023 | Association of epidural analgesia during labor and early postpartum urinary incontinence among women delivered vaginally | Excluded | Excluded by title and abstract | TB | 2024/07/05-2024/08/16 | Not eligibility | Google Scholar |
|  | Barbosa-Silva J etal,2024 | Relationship of female pelvic floor muscle function and body composition: cross-sectional study | Excluded | Excluded by title and abstract | TB | 2024/07/05-2024/08/16 | Not eligibility | Google Scholar |
|  | Shanshan H etal,2024 | Prevalence of lumbopelvic pain during pregnancy: A systematic review and meta‐analysis of cross‐sectional studies | Excluded | Excluded by title and abstract | TB | 2024/07/05-2024/08/16 | Not eligibility | Google Scholar |
|  | Xu L etal,2024 | The genetic architecture and evolutionary consequences of the human pelvic form | Excluded | Excluded by title and abstract | TB | 2024/07/05-2024/08/16 | Not eligibility | Google Scholar |
|  | Verghese T,2021 | Pelvic organ prolapses and oestrogen | Excluded | Excluded by title and abstract | TB | 2024/07/05-2024/08/16 | Not eligibility | Google Scholar |
|  | Hoque AM etal,2021 | Incidence, trends and risk factors for perineal injuries of low-risk pregnant women: Experience from a midwife run obstetric unit, South Africa | Excluded | Excluded by title and abstract | TB | 2024/07/05-2024/08/16 | Not eligibility | Google Scholar |
|  | Samir Dawood S,2021 | Relationship between Post-menopausal women’s knowledge about pelvic organ prolapse and their autonomy preference | Excluded | Excluded by title and abstract | TB | 2024/07/05-2024/08/16 | Not eligibility | Google Scholar |
|  | Wood SN etal, 2022 | A scoping review on women’s sexual health in the postpartum period | Excluded | Excluded by title and abstract | TB | 2024/07/05-2024/08/16 | Not eligibility | Google Scholar |
|  | Marcos S etal,2024 | Prevalence of diastasis recti abdominis in postmenopausal women with stress urinary incontinence | Excluded | Excluded by title and abstract | TB | 2024/07/05-2024/08/16 | Not eligibility | Google Scholar |
|  | Bazzoun Y etal,2021 | Chronic vulvar pain after female genital mutilation/cutting: a retrospective study | Excluded | Excluded by title and abstract | TB | 2024/07/05-2024/08/16 | Not eligibility | Google Scholar |
|  | Ramadan Hassan SG etal,2020 | Effect of Kegel Exercise on Improving Manifestations of Uterine Prolapse among Pre menopausal Women | Excluded | Excluded by title and abstract | TB | 2024/07/05-2024/08/16 | Not eligibility | Google Scholar |
|  | Kunkel GM etal,2020 | Prophylaxis in Pelvic Floor Surgery | Excluded | Excluded by title and abstract | TB | 2024/07/05-2024/08/16 | Not eligibility | Google Scholar |
|  | Endalifer ML and Diress G,2021 | Effect of overweight/obesity on caesarean section occurrence among reproductive-aged women in Ethiopia | Excluded | Excluded by title and abstract | TB | 2024/07/05-2024/08/16 | Not eligibility | Google Scholar |
|  | Shehata Ibrahim S etal,2023 | Knowledge and Practices of Women Regarding Risk Factors and Preventive Measures of Vaginal Prolapse | Excluded | Excluded by title and abstract | TB | 2024/07/05-2024/08/16 | Not eligibility | Google Scholar |
|  | Troko J etal,2016 | Predicting urinary incontinence in women in later life: A systematic review | Excluded | Excluded by title and abstract | TB | 2024/07/05-2024/08/16 | Not eligibility | Google Scholar |
|  | Zeleke BM etal,2016 | Symptomatic pelvic floor disorders in community-dwelling older Australian women | Excluded | Excluded by title and abstract | TB | 2024/07/05-2024/08/16 | Not eligibility | Google Scholar |
|  | Madu TO etal,2023 | Knowledge and Practice of Pelvic Floor Muscle Exercises Among Antenatal and Postnatal Women Attending a Secondary Health Facility in Southeast, Nigeria. | Excluded | Excluded by title and abstract | TB | 2024/07/05-2024/08/16 | Not eligibility | Google Scholar |
|  | Abdel-Wahab Afifi Araby Ali O etal,2024 | Effect of Spontaneous Open-glottis versus Valsalva Closed-glottis Pushing during Second Stage of Labour on Pelvic Floor Morbidity and Fatigue | Excluded | Excluded by title and abstract | TB | 2024/07/05-2024/08/16 | Not eligibility | Google Scholar |
|  | Kueh YC,2018 | Urinary incontinence among pregnant women attending an antenatal clinic at a tertiary teaching hospital in North-East Malaysia | Excluded | Excluded by title and abstract | TB | 2024/07/05-2024/08/16 | Not eligibility | Google Scholar |
|  | Ayenew AA,2021 | Incidence, causes, and maternofetal outcomes of obstructed labor in Ethiopia: systematic review and meta-analysis | Excluded | Excluded by title and abstract | TB | 2024/07/05-2024/08/16 | Not eligibility | Google Scholar |
|  | Gjerde JL etal ,2018 | Life after pelvic organ prolapse surgery: a qualitative study in Amhara region, Ethiopia | Excluded | Excluded by title and abstract | TB | 2024/07/05-2024/08/16 | Not eligibility | Google Scholar |
|  | Lin KY etal,2023 | Analgesic Efficacy of Acupuncture on Chronic Pelvic Pain: A Systemic Review and Meta-Analysis Study | Excluded | Excluded by title and abstract | TB | 2024/07/05-2024/08/16 | Not eligibility | Google Scholar |
|  | Kayondo M etal,2021 | Impact of surgery on quality of life of Ugandan women with symptomatic pelvic organ prolapse: a prospective cohort study | Excluded | Excluded by title and abstract | TB | 2024/07/05-2024/08/16 | Not eligibility | Google Scholar |
|  | Addisu D etal,2023 | The prevalence of pelvic organ prolapse and associated factors in Ethiopia: a systematic review and meta-analysis | Excluded | Excluded by title and abstract | TB | 2024/07/05-2024/08/16 | Not eligibility | Google Scholar |
|  | Azadi A etal,2022 | Female Pelvic Medicine & Reconstructive Surgery | Excluded | Excluded by title and abstract | TB | 2024/07/05-2024/08/16 | Not eligibility | Google Scholar |
|  | Karout S etal,2021 | Prevalence, risk factors, and management practices of primary dysmenorrhea among young females | Excluded | Excluded by title and abstract | TB | 2024/07/05-2024/08/16 | Not eligibility | Google Scholar |
|  | Thapa B etal, 2014 | Contributing factors of utero-vaginal prolapse among women attending in Bharatpur hospital | Excluded | Excluded by title and abstract | TB | 2024/07/05-2024/08/16 | Not eligibility | Google Scholar |
|  | Tugume R etal,2021 | Prevalence and factors associated with pelvic organ prolapse among women attending the gynecology outpatient clinic at a tertiary hospital in southwestern Uganda | Excluded | Excluded by title and abstract | TB | 2024/07/05-2024/08/16 | Not eligibility | Google Scholar |
|  | Jokhio AH etal,2014 | Prevalence of obstetric fistula: a population‐based study in rural P akistan | Excluded | Excluded by title and abstract | TB | 2024/07/05-2024/08/16 | Not eligibility | Google Scholar |
|  | Gurland B etal,2018 | A collaborative approach to multicompartment pelvic organ prolapse | Excluded | Excluded by title and abstract | TB | 2024/07/05-2024/08/16 | Not eligibility | Google Scholar |
|  | Shrestha S etal,2016 | Urinary complication following cystocele repair in pelvic organ prolapse | Excluded | Excluded by title and abstract | TB | 2024/07/05-2024/08/16 | Not eligibility | Google Scholar |
|  | Segarra AC,2024 | Conservative Treatment of Female Pelvic Organ Prolapse | Excluded | Excluded by title and abstract | TB | 2024/07/05-2024/08/16 | Not eligibility | Google Scholar |
|  | Faisal‐Cury A etal,2013 | The relationship between depressive/anxiety symptoms during pregnancy/postpartum and sexual life decline after delivery | Excluded | Excluded by title and abstract | TB | 2024/07/05-2024/08/16 | Not eligibility | Google Scholar |
|  | Abrams P etal,2018 | evaluation and treatment of urinary incontinence, pelvic organ prolapse and faecal incontinence | Excluded | Excluded by title and abstract | TB | 2024/07/05-2024/08/16 | Not eligibility | Google Scholar |
|  | Law H,and Fiadjoe P,2012 | Urogynaecological problems in pregnancy | Excluded | Excluded by title and abstract | TB | 2024/07/05-2024/08/16 | Not eligibility | Google Scholar |
|  | Mazumder AA etal,2022 | Use of Silicon Vaginal Pessary for Treatment of Pelvic Organ Prolapse | Excluded | Excluded by title and abstract | TB | 2024/07/05-2024/08/16 | Not eligibility | Google Scholar |
|  | O’Shea M etal,2022 | Prevalence of lower urinary tract symptoms in a cohort of Australian servicewomen and female veterans | Excluded | Excluded by title and abstract | TB | 2024/07/05-2024/08/16 | Not eligibility | Google Scholar |
|  | Schulten SF. 2022 | Risk factors for pelvic organ prolapse recurrence after sacrospinous hysteropexy or vaginal hysterectomy with uterosacral ligament suspension | Excluded | Excluded by title and abstract | TB | 2024/07/05-2024/08/16 | Not eligibility | Google Scholar |
|  | Aitbayeva B etal,2022 | Effects of unilateral apical sling and laparoscopic sacrocolpopexy on the outcome in women with apical prolapse | Excluded | Excluded by title and abstract | TB | 2024/07/05-2024/08/16 | Not eligibility | Google Scholar |
|  | Koyuncu A etal,2020 | Heavy load carrying and adverse reproductive health among women in Tanzania and Nepal | Excluded | Excluded by title and abstract | TB | 2024/07/05-2024/08/16 | Not eligibility | Google Scholar |
|  | ALdeen E,2015 | Impact of Different Degrees of Uterine Prolapse on Sexual Function of Women at Teaching Hospitals in Al-Hilla City. | Excluded | Excluded by title and abstract | TB | 2024/07/05-2024/08/16 | Not eligibility | Google Scholar |
|  | Azadi A etal,2021 | Feasibility of Risk Reducing Salpingo-Oophorectomy at the Time of Abdominal Surgery for Correction of Pelvic Organ Prolapse and Urinary Incontinence | Excluded | Excluded by title and abstract | TB | 2024/07/05-2024/08/16 | Not eligibility | Google Scholar |
|  | Younus SK, and Al-Dabakh SR,2017 | Rate of pelvic organ prolapse in Maternity Teaching Hospital in Erbil city | Excluded | Excluded by title and abstract | TB | 2024/07/05-2024/08/16 | Not eligibility | Google Scholar |
|  | Mahishale A, and Bhattarai A,2023 | Prevalence of Pelvic Crossed Syndrome in Females with Primary Dysmenorrhea and its Impact on Physical Activity | Excluded | Excluded by title and abstract | TB | 2024/07/05-2024/08/16 | Not eligibility | Google Scholar |
|  | Krause HG etal,2017 | Mental health screening in women with severe pelvic organ prolapse, chronic fourth-degree obstetric tear and genital tract fistula in western Uganda | Excluded | Excluded by title and abstract | TB | 2024/07/05-2024/08/16 | Not eligibility | Google Scholar |
|  | Getie Mekonnen E etal,2021 | Sexual dysfunction among men with diabetes mellitus attending chronic out-patient department at the three hospitals of Northwest Amhara region, Ethiopia | Excluded | Excluded by title and abstract | TB | 2024/07/05-2024/08/16 | Not eligibility | Google Scholar |
|  | Pope R etal,2018 | Sexual function before and after vesicovaginal fistula repair | Excluded | Excluded by title and abstract | TB | 2024/07/05-2024/08/16 | Not eligibility | Google Scholar |
|  | Widyasari A etal,2024 | Collagen-1 and elastin expression in cervical tissue: A comparison across cervical elongation, pelvic organ prolapse, and combined conditions | Excluded | Excluded by title and abstract | TB | 2024/07/05-2024/08/16 | Not eligibility | Google Scholar |
|  | Jinapun P, and Sangnucktham T,2024 | Effects of pelvic floor muscle training on urinary incontinence during the third trimester of nulliparous pregnant women | Excluded | Excluded by title and abstract | TB | 2024/07/05-2024/08/16 | Not eligibility | Google Scholar |
|  | Ali HT etal,2022 | Epidemiology and risk factors for ovarian cancer | Excluded | Excluded by title and abstract | TB | 2024/07/05-2024/08/16 | Not eligibility | Google Scholar |
|  | Belayneh T etal,2021 | Pelvic organ prolapse surgery and health-related quality of life: a follow-up study | Excluded | Excluded by title and abstract | TB | 2024/07/05-2024/08/16 | Not eligibility | Google Scholar |
|  | Linder KE etal,2021 | Treatment patterns in women seeking care for endometriosis at an endometriosis center | Excluded | Excluded by title and abstract | TB | 2024/07/05-2024/08/16 | Not eligibility | Google Scholar |
|  | Krause H ,2021 | A multidisciplinary approach is needed to improve mental health and social connectedness in women suffering with obstetric fistula, chronic 4th degree tear and severe pelvic organ prolapse in limited resource regions | Excluded | Excluded by title and abstract | TB | 2024/07/05-2024/08/16 | Not eligibility | Google Scholar |
|  | Mao M etal,2024 | Effect of generalized anxiety disorders on the success of pessary treatment for pelvic organ prolapse | Excluded | Excluded by title and abstract | TB | 2024/07/05-2024/08/16 | Not eligibility | Google Scholar |
|  | Sward L etal,2023 | Pelvic girdle pain in pregnancy: a review | Excluded | Excluded by title and abstract | TB | 2024/07/05-2024/08/16 | Not eligibility | Google Scholar |
|  | Soloveva OV etal,2022 | Analysis of risk factors for pelvic organ prolapse in females after hysterectomy | Excluded | Excluded by title and abstract | TB | 2024/07/05-2024/08/16 | Not eligibility | Google Scholar |
|  | Mubashir H etal,2022 | The Risk Factors and Incidence of Perineal Tears among Pregnant Women | Excluded | Excluded by title and abstract | TB | 2024/07/05-2024/08/16 | Not eligibility | Google Scholar |
|  | Yi Z, and Romainoor N,2023 | A Systematic Literature Review for Interface Design of Pelvic Floor Muscle Training Mobile App base on mHealth 2017-2022 | Excluded | Excluded by title and abstract | TB | 2024/07/05-2024/08/16 | Not eligibility | Google Scholar |
|  | Moon H etal,2021 | Interaction and main effects of physical and depressive symptoms on quality of life in Korean women seeking care for rectal prolapse | Excluded | Excluded by title and abstract | TB | 2024/07/05-2024/08/16 | Not eligibility | Google Scholar |
|  | Wall LL etal,2021 | The Sims position and the Sims vaginal speculum, re-examined | Excluded | Excluded by title and abstract | TB | 2024/07/05-2024/08/16 | Not eligibility | Google Scholar |
|  | ROBERT Z, 2021 | Diagnosis and Management of Pelvic Floor Dyssynergia | Excluded | Excluded by title and abstract | TB | 2024/07/05-2024/08/16 | Not eligibility | Google Scholar |
|  | Al Hassan RM etal,2024 | Evaluation of Electromagnetic Therapy in the Treatment of Severe Dysmenorrhea in Young Women of Basrah | Excluded | Excluded by title and abstract | TB | 2024/07/05-2024/08/16 | Not eligibility | Google Scholar |
|  | Ahmad N etal,2024 | A review of functional pelvic floor imaging modalities and their effectiveness | Excluded | Excluded by title and abstract | TB | 2024/07/05-2024/08/16 | Not eligibility | Google Scholar |
|  | Nakimuli A etal,2014 | Pregnancy, parturition and preeclampsia in women of African ancestry. American journal of obstetrics and gynecology | Excluded | Excluded by title and abstract | TB | 2024/07/05-2024/08/16 | Not eligibility | Google Scholar |
|  | Zhao Y etal,2024 | Pelvic floor parameters predict postpartum stress urinary incontinence | Excluded | Excluded by title and abstract | TB | 2024/07/05-2024/08/16 | Not eligibility | Google Scholar |
|  | Ismail A etal,2024 | Understanding Awareness, Perspectives and Habits in Pakistani Women of Reproductive Age | Excluded | Excluded by title and abstract | TB | 2024/07/05-2024/08/16 | Not eligibility | Google Scholar |
|  | Lin M etal,2020 | Tissue-engineered repair material for pelvic floor dysfunction | Excluded | Excluded by title and abstract | TB | 2024/07/05-2024/08/16 | Not eligibility | Google Scholar |
|  | Sørensen HJ,2019 | Associations between symptoms of depression and sociodemographic factors and pregnancy-related complaints among pregnant women in urban and rural Nepal | Excluded | Excluded by title and abstract | TB | 2024/07/05-2024/08/16 | Not eligibility | Google Scholar |
|  | Dunn G etal,2019 | Trajectories of lower back, upper back, and pelvic girdle pain during pregnancy and early postpartum in primiparous women | Excluded | Excluded by title and abstract | TB | 2024/07/05-2024/08/16 | Not eligibility | Google Scholar |
|  | Mammo M etal,2022 | Prevalence of primary dysmenorrhea, its intensity and associated factors among female students at high schools of Wolaita Zone, Southern Ethiopia | Excluded | Excluded by title and abstract | TB | 2024/07/05-2024/08/16 | Not eligibility | Google Scholar |
|  | Kirby AC etal,2012 | An update on the current and future demand for care of pelvic floor disorders in the United States | Excluded | Excluded by title and abstract | TB | 2024/07/05-2024/08/16 | Not eligibility | Google Scholar |
|  | Omeke CA, and Azuka CE,2023 | Urinary incontinence among women in sub-Saharan Africa–an overview | Excluded | Excluded by title and abstract | TB | 2024/07/05-2024/08/16 | Not eligibility | Google Scholar |
|  | Bakhtiar K etal,2013 | Prevalence of nocturnal enuresis and its associated factors in primary school and preschool children of Khorramabad in 2013 | Excluded | Excluded by title and abstract | TB | 2024/07/05-2024/08/16 | Not eligibility | Google Scholar |
|  | Hannon S etal , 2023 | Physical health and comorbid anxiety and depression across the first year postpartum in Ireland | Excluded | Excluded by title and abstract | TB | 2024/07/05-2024/08/16 | Not eligibility | Google Scholar |
|  | Shayo BC etal,2019 | Vaginal pessaries in the management of symptomatic pelvic organ prolapse in rural Kilimanjaro, Tanzania | Excluded | Excluded by title and abstract | TB | 2024/07/05-2024/08/16 | Not eligibility | Google Scholar |
|  | Garcia AN etal,2024 | Spanish-Speaking Latinas with Pelvic Floor Disorders | Excluded | Excluded by title and abstract | TB | 2024/07/05-2024/08/16 | Not eligibility | Google Scholar |
|  | Böhmler RL,2024 | Cesarean delivery on maternal request | Excluded | Excluded by title and abstract | TB | 2024/07/05-2024/08/16 | Not eligibility | Google Scholar |
|  | Anees S etal,2021 | Elements Affecting Adherence To Pelvic Floor Rehabilitation Exercises In Females With Urinary Incontinence | Excluded | Excluded by title and abstract | TB | 2024/07/05-2024/08/16 | Not eligibility | Google Scholar |
|  | Saeed J, and Abdullah L, 2022 | Knowledge, attitudes, and practices of pelvic floor muscle exercises among pregnant women visiting public hospitals | Excluded | Excluded by title and abstract | TB | 2024/07/05-2024/08/16 | Not eligibility | Google Scholar |
|  | Gresty H etal, 2023 | Overview, epidemiology, and etiopathogenetic differences in urogenital fistulae in the resourced and resource-limited worlds | Excluded | Excluded by title and abstract | TB | 2024/07/05-2024/08/16 | Not eligibility | Google Scholar |
|  | Paudel S etal,2019 | Efficacy and safety of Aspirin plus intermittent pneumatic compression device as thromboprophylaxis after total hip arthroplasty | Excluded | Excluded by title and abstract | TB | 2024/07/05-2024/08/16 | Not eligibility | Google Scholar |
|  | Bazi T ,2024 | What Type of Review is Yours?. International Urogynecology Journal | Excluded | Excluded by title and abstract | TB | 2024/07/05-2024/08/16 | Not eligibility | Google Scholar |
|  | Hoque AM, and Buckus S. 2021 | Prevalence, Seroconversion and Incidence of Maternal HIV during Pregnancy. | Excluded | Excluded by title and abstract | TB | 2024/07/05-2024/08/16 | Not eligibility | Google Scholar |
|  | Wahab etal,2023 | Frequency of Diastasis Recti Abdominis in Pregnant Women of Third Trimester | Excluded | Excluded by title and abstract | TB | 2024/07/05-2024/08/16 | Not eligibility | Google Scholar |
|  | Mustafa I etal,2020 | novel treatment approach for women with chronic pelvic pain syndrome leading to increased pelvic functionality | Excluded | Excluded by title and abstract | TB | 2024/07/05-2024/08/16 | Not eligibility | Google Scholar |
|  | Brunelli WS etal,2024 | Repercussions of perineal repair using surgical glue or suture thread on postpartum outcomes: A controlled randomized clinical trial in São Paulo, Brazil | Excluded | Excluded by title and abstract | TB | 2024/07/05-2024/08/16 | Not eligibility | Google Scholar |
|  | Allen-Brady K etal,2020 | Risk of pelvic organ prolapse treatment based on extended family history | Excluded | Excluded by title and abstract | TB | 2024/07/05-2024/08/16 | Not eligibility | Google Scholar |
|  | Barcikowska Z etal,2020 | Urine biomarkers in the management of adult neurogenic lower urinary tract dysfunction | Excluded | Excluded by title and abstract | TB | 2024/07/05-2024/08/16 | Not eligibility | Google Scholar |
|  | Karim R etal,2019 | Incontinence of urine in pregnant women. Journal of Postgraduate Medical Institute | Excluded | Excluded by title and abstract | TB | 2024/07/05-2024/08/16 | Not eligibility | Google Scholar |
|  | Krause HG etal,2022 | Urinary Incontinence Following Obstetric Fistula Surgery | Excluded | Excluded by title and abstract | TB | 2024/07/05-2024/08/16 | Not eligibility | Google Scholar |
|  | Moss C etal,2023 | Analysing changes to the flow of public funding within local health and care systems | Excluded | Excluded by title and abstract | TB | 2024/07/05-2024/08/16 | Not eligibility | Google Scholar |
|  | Dingeta T etal,2019 | Unmet need for contraception among young married women in eastern Ethiopia | Excluded | Excluded by title and abstract | TB | 2024/07/05-2024/08/16 | Not eligibility | Google Scholar |
|  | Torstensson T etal,2018 | Anatomical landmarks of the intra-pelvic side-wall as sources of pain in women with and without pregnancy-related chronic pelvic pain after childbirth | Excluded | Excluded by title and abstract | TB | 2024/07/05-2024/08/16 | Not eligibility | Google Scholar |
|  | Ungure A etal,2022 | 167 Analysis of child-birth related risk factors for postpartum endometritis in riga maternity hospital, Latvia | Excluded | Excluded by title and abstract | TB | 2024/07/05-2024/08/16 | Not eligibility | Google Scholar |
|  | Yuniarti F and Ivantarina D,2022 | Literature Review: Komplikasi Maternal dan Neonatal Akibat Persalinan Macet | Excluded | Excluded by title and abstract | TB | 2024/07/05-2024/08/16 | Not eligibility | Google Scholar |
|  | Othman A,2024 | The effects of pregnancy and childbirth on women's health-related quality of life | Excluded | Excluded by title and abstract | TB | 2024/07/05-2024/08/16 | Not eligibility | Google Scholar |
|  | Shalaby Awad Mahmoud N etal,2024 | Effect of the Standard Breast Crawl Technique on Initiation of Breastfeeding and Maternal Outcomes | Excluded | Excluded by title and abstract | TB | 2024/07/05-2024/08/16 | Not eligibility | Google Scholar |
|  | Talebi E etal,2024 | Performance evaluation of novel safflower (Carthamus tinctorius L.) genotypes under salinity stress conditions | Excluded | Excluded by title and abstract | TB | 2024/07/05-2024/08/16 | Not eligibility | Google Scholar |
|  | Mutiara FR etal,2022 | The Prevalence of Back Pain and the Pattern of Complaints in Pregnant Women at the Gatak Community Health Centers | Excluded | Excluded by title and abstract | TB | 2024/07/05-2024/08/16 | Not eligibility | Google Scholar |
|  | Begum S,2022 | Attitudes and associated factors to antenatal physiotherapy in pregnant women at selected Hospitals in Bangladesh | Excluded | Excluded by title and abstract | TB | 2024/07/05-2024/08/16 | Not eligibility | Google Scholar |
|  | Woldegeorgis BZ etal,2022 | Episiotomy practice and its associated factors in Africa: A systematic review and meta-analysis | Excluded | Excluded by title and abstract | TB | 2024/07/05-2024/08/16 | Not eligibility | Google Scholar |
|  | Hu P etal,2023 | Association between heavy metal exposures and the prevalence of pelvic inflammatory disease | Excluded | Excluded by title and abstract | TB | 2024/07/05-2024/08/16 | Not eligibility | Google Scholar |
|  | Milka W etal,,2023 | Antenatal perineal massage-risk of perineal injuries, pain, urinary incontinence and dyspereunia-a systematic review | Excluded | Excluded by title and abstract | TB | 2024/07/05-2024/08/16 | Not eligibility | Google Scholar |
|  | Diop B etal,2018 | Management of Genital Prolapse: Experience of One African Surgery Department | Excluded | Excluded by title and abstract | TB | 2024/07/05-2024/08/16 | Not eligibility | Google Scholar |
|  | Sharma S etal,2023 | Three dimensional printing of deformed ankle foot and pelvis using poly lactic acid for pre surgical planning | Excluded | Excluded by title and abstract | TB | 2024/07/05-2024/08/16 | Not eligibility | Google Scholar |
|  | Blanco Gutiérrez V,2023 | The experience of women from underrepresented groups with urinary incontinence | Excluded | Excluded by title and abstract | TB | 2024/07/05-2024/08/16 | Not eligibility | Google Scholar |
|  | Imoto A etal,2021 | Health-related quality of life in parous women with pelvic organ prolapse and/or urinary incontinence in Bangladesh | Excluded | Excluded by title and abstract | TB | 2024/07/05-2024/08/16 | Not eligibility | Google Scholar |
|  | Alshiakh F,2022 | The lived experience of pelvic organ prolapse in Saudi Arabia | Excluded | Excluded by title and abstract | TB | 2024/07/05-2024/08/16 | Not eligibility | Google Scholar |
|  | Chunmei D etal,2023 | Self-efficacy associated with regression from pregnancy-related pelvic girdle pain and low back pain following pregnancy | Excluded | Excluded by title and abstract | TB | 2024/07/05-2024/08/16 | Not eligibility | Google Scholar |
|  | Unai E, and Yagmur Y,2023 | Determining the Effect of Prolapse Stages on Quality of Life | Excluded | Excluded by title and abstract | TB | 2024/07/05-2024/08/16 | Not eligibility | Google Scholar |
|  | Romanzi L etal,2012 | Hysteropexy compared to hysterectomy for uterine prolapse surgery | Excluded | Excluded by title and abstract | TB | 2024/07/05-2024/08/16 | Not eligibility | Google Scholar |
|  | Spitznagle T ,2022 | Physical Therapy for Women with Obstetric Fistula | Excluded | Excluded by title and abstract | TB | 2024/07/05-2024/08/16 | Not eligibility | Google Scholar |
|  | Yang M etal,2023 | Finite Element Analysis of the Normal Female Pelvic Floor Structure | Excluded | Excluded by title and abstract | TB | 2024/07/05-2024/08/16 | Not eligibility | Google Scholar |
|  | Al-Falahi MA,2024 | A Rare Case of Focal Renal Fibromuscular Dysplasia Treated With Angioplasty | Excluded | Excluded by title and abstract | TB | 2024/07/05-2024/08/16 | Not eligibility | Google Scholar |
|  | Norton JM etal,2017 | Nonbiologic factors that impact management in women with urinary incontinence: review of the literature and findings from a National Institute of Diabetes and Digestive and Kidney Diseases workshop | Excluded | Excluded by title and abstract | TB | 2024/07/05-2024/08/16 | Not eligibility | Google Scholar |
|  | Ndoye M etal,2023 | Female Genital Mutilation/Cutting. InFemale Genitourinary and Pelvic Floor Reconstruction | Excluded | Excluded by title and abstract | TB | 2024/07/05-2024/08/16 | Not eligibility | Google Scholar |
|  | Jonga T etal,2024 | Prevalence of low back pain and associated factors among bank workers at Hawassa city, Northern Zone, Sidama Region, Southern Ethiopia | Excluded | Excluded by title and abstract | TB | 2024/07/05-2024/08/16 | Not eligibility | Google Scholar |
|  | Giri S,2023 | Knowledge Regarding Uterine Prolapse Among Reproductive Age Group Women of Birendranagar, Surkhet, Nepal | Excluded | Excluded by title and abstract | TB | 2024/07/05-2024/08/16 | Not eligibility | Google Scholar |
|  | Lussiez A etal,2022 | A multi-modal study examining long-term bowel, urinary, and sexual function after rectal cancer surgery | Excluded | Excluded by title and abstract | TB | 2024/07/05-2024/08/16 | Not eligibility | Google Scholar |
|  | Sharma C etal,2014 | Fecal evacuation disorder among patients with solitary rectal ulcer syndrome: a case-control study | Excluded | Excluded by title and abstract | TB | 2024/07/05-2024/08/16 | Not eligibility | Google Scholar |
|  | Moon H etal, 2021 | Interaction and main effects of physical and depressive symptoms on quality of life in Korean women seeking care for rectal prolapse | Excluded | Excluded by title and abstract | TB | 2024/07/05-2024/08/16 | Not eligibility | Google Scholar |
|  | Yacubovich Yetal,2019 | The prevalence of primary dysmenorrhea among students and its association with musculoskeletal and myofascial pain | Excluded | Excluded by title and abstract | TB | 2024/07/05-2024/08/16 | Not eligibility | Google Scholar |
|  | Meierhofer R etal,2022 | Water carrying in hills of Nepal–associations with women’s musculoskeletal disorders, uterine prolapse, and spontaneous abortions | Excluded | Excluded by title and abstract | TB | 2024/07/05-2024/08/16 | Not eligibility | Google Scholar |
|  | Amza M etal,2024 | Dysmenorrhea and Its Impact on Patients’ Quality of Life | Excluded | Excluded by title and abstract | TB | 2024/07/05-2024/08/16 | Not eligibility | Google Scholar |
|  | Pant U etal, 2028 | Risk profile of uterovaginal prolapse | Excluded | Excluded by title and abstract | TB | 2024/07/05-2024/08/16 | Not eligibility | Google Scholar |
|  | Hoque SS etal,2023 | Comparing patient-reported outcome measures for pain in women with pelvic floor disorders pre-and post-surgical management | Excluded | Excluded by title and abstract | TB | 2024/07/05-2024/08/16 | Not eligibility | Google Scholar |
|  | Keyser L etal,2021 | Rehabilitative care practices in the management of childbirth-related pelvic fistula | Excluded | Excluded by title and abstract | TB | 2024/07/05-2024/08/16 | Not eligibility | Google Scholar |
|  | Shayo BC etal,2020 | Management of stress urinary incontinence using vaginal incontinence pessaries in rural Kilimanjaro, Tanzania | Excluded | Excluded by title and abstract | TB | 2024/07/05-2024/08/16 | Not eligibility | Google Scholar |
|  | Lian WQ etal,2019 | Constipation and risk of urinary incontinence in women | Excluded | Excluded by title and abstract | TB | 2024/07/05-2024/08/16 | Not eligibility | Google Scholar |
|  | Rostami-Moez M etal,2023 | Examining the health-related needs of females during menopause | Excluded | Excluded by title and abstract | TB | 2024/07/05-2024/08/16 | Not eligibility | Google Scholar |
|  | Armour M etal,2019 | Endometriosis and chronic pelvic pain have similar impact on women, but time to diagnosis is decreasing | Excluded | Excluded by title and abstract | TB | 2024/07/05-2024/08/16 | Not eligibility | Google Scholar |
|  | Hameed S etal,2023 | Frequency of Infected Episiotomy in Primigravida Women After Vaginal Delivery | Excluded | Excluded by title and abstract | TB | 2024/07/05-2024/08/16 | Not eligibility | Google Scholar |
|  | OUEDRAOGO I, and AYENACHEW F,2021 | Contemporary Issues in Obstetric Fistula | Excluded | Excluded by title and abstract | TB | 2024/07/05-2024/08/16 | Not eligibility | Google Scholar |
|  | Taak RH etal,2024 | Awareness and Motivation towards Gynecological Physical Therapy | Excluded | Excluded by title and abstract | TB | 2024/07/05-2024/08/16 | Not eligibility | Google Scholar |
|  | Ekwedigwe KC etal,2018 | Prevalence and antimicrobial susceptibility of asymptomatic bacteriuria among women with pelvic organ prolapse in Abakaliki, South-East Nigeria | Excluded | Excluded by title and abstract | TB | 2024/07/05-2024/08/16 | Not eligibility | Google Scholar |
|  | Seif Mohammed Elattar M etal,2022 | Educational program for multipara women with stress urinary incontinence | Excluded | Excluded by title and abstract | TB | 2024/07/05-2024/08/16 | Not eligibility | Google Scholar |
|  | Witkoś J etal,2021 | Level of knowledge among physiotherapy students concerning the management of stress urinary incontinence in women | Excluded | Excluded by title and abstract | TB | 2024/07/05-2024/08/16 | Not eligibility | Google Scholar |
|  | Hall B etal,2021 | Design of programs to train pelvic floor muscles in men with urinary dysfunction: systematic review | Excluded | Excluded by title and abstract | TB | 2024/07/05-2024/08/16 | Not eligibility | Google Scholar |
|  | Gjerde Jl,2012 | Female urinary incontinence: perceptions and practice. A qualitative study from Amhara Region, Ethiopia | Excluded | Excluded by title and abstract | TB | 2024/07/05-2024/08/16 | Not eligibility | Google Scholar |
|  | Chawanpaiboon S etal,2023 | Maternal complications and risk factors associated with assisted vaginal delivery | Excluded | Excluded by title and abstract | TB | 2024/07/05-2024/08/16 | Not eligibility | Google Scholar |
|  | Abza MW, 2022 | Cost-effectiveness Analysis of Surgical Repair for Obstetric Fistula in Ethiopia | Excluded | Excluded by title and abstract | TB | 2024/07/05-2024/08/16 | Not eligibility | Google Scholar |
|  | Emasu A etal,2019 | Reintegration needs of young women following genitourinary fistula surgery in Uganda | Excluded | Excluded by title and abstract | TB | 2024/07/05-2024/08/16 | Not eligibility | Google Scholar |
|  | Pesikhani MD etal,2023 | Assessment of manometric results following posterior pericervical repair or level I to III surgical procedures | Excluded | Excluded by title and abstract | TB | 2024/07/05-2024/08/16 | Not eligibility | Google Scholar |
|  | Chang TP, and Ho ZP,2016 | Pregnant Women-A Smooth Birth of a Child-A Comparison Study | Excluded | Excluded by title and abstract | TB | 2024/07/05-2024/08/16 | Not eligibility | Google Scholar |
|  | Martire FG etal,2023 | Early noninvasive diagnosis of endometriosis: Dysmenorrhea and specific ultrasound findings are important indicators in young women | Excluded | Excluded by title and abstract | TB | 2024/07/05-2024/08/16 | Not eligibility | Google Scholar |
|  | Memon HU etal,2013 | Vaginal childbirth and pelvic floor disorders | Excluded | Excluded by title and abstract | TB | 2024/07/05-2024/08/16 | Not eligibility | Google Scholar |
|  | Jorge CH etal, 2024 | Pelvic floor muscle training as treatment for female sexual dysfunction: a systematic review and meta-analysis | Excluded | Excluded by title and abstract | WN | 2024/07/05-2024/08/16 | Not eligible | Scopus |
|  | Fernández MJ etal,2024 | Oocyte collection and outcome following oncologic treatment | Excluded | Excluded by title and abstract | WN | 2024/07/05-2024/08/16 | Not eligible | Scopus |
|  | Ralphsmith M etal,2024 | Development of a conceptual framework for a new patient-reported outcome measure for pain in women following mesh surgery for pelvic floor disorders | Excluded | Excluded by title and abstract | WN | 2024/07/05-2024/08/16 | Not eligible | Scopus |
|  | Sologuren G etal,2023 | Qualitative analysis of the sexuality in people with acquired motor disability | Excluded | Excluded by title and abstract | WN | 2024/07/05-2024/08/16 | Not eligible | Scopus |
|  | Brunelli WS etal ,2024 | Repercussions of perineal repair using surgical glue or suture thread on postpartum outcomes | Excluded | Excluded by title and abstract | WN | 2024/07/05-2024/08/16 | Not eligible | Scopus |
|  | Kayembe AT etal ,2024 | Factors associated with pelvic organ prolapse: case-control study in two hospitals of Bon-Berger and Saint Georges of the city of Kananga in the Democratic Republic of the Congo | Excluded | Excluded by title and abstract | WN | 2024/07/05-2024/08/16 | Not eligible | Scopus |
|  | Kefeni M etal ,2024 | Magnitude and factors associated with catheter associated urinary tract infection, and antimicrobial susceptibility profile at Hawassa, Sidama Regional State, Ethiopia | Excluded | Excluded by title and abstract | WN | 2024/07/05-2024/08/16 | Not eligible | Scopus |
|  | Mashayekh Amiri S etal ,2023 | Examining psychometric properties of the Iranian version of exclusive breastfeeding social support scale | Excluded | Excluded by title and abstract | WN | 2024/07/05-2024/08/16 | Not eligible | Scopus |
|  | Peinado-Molina RA etal ,2023 | Pelvic floor dysfunction: prevalence and associated factors | Excluded | Excluded by title and abstract | WN | 2024/07/05-2024/08/16 | Not eligible | Scopus |
|  | Kurniawati EM etal ,2023 | Differences between 25-hydroxyvitamin D levels in patients with pelvic organ prolapse and non-pelvic organ prolapse: A systematic review | Excluded | Excluded by title and abstract | WN | 2024/07/05-2024/08/16 | Not eligible | Scopus |
|  | Siyoum M etal ,2023 | Reliability and validity of the Sidaamu Afoo version of the pelvic organ prolapse symptom score questionnaire | Excluded | Excluded by title and abstract | WN | 2024/07/05-2024/08/16 | Not eligible | Scopus |
|  | Zhang W etal ,2023 | Role of neuroinflammation in neurodegeneration development | Excluded | Excluded by title and abstract | WN | 2024/07/05-2024/08/16 | Not eligible | Scopus |
|  | Shitu AW etal, 2023 | Delay in seeking treatment and associated factors among women with pelvic organ prolapse in Wolaita zone, Southern Ethiopia | Excluded | Excluded by title and abstract | WN | 2024/07/05-2024/08/16 | Not eligible | Scopus |
|  | Camacho S etal ,2023 | Sexual dysfunction worsens both the general and specific quality of life of women with irritable bowel syndrome | Excluded | Excluded by title and abstract | WN | 2024/07/05-2024/08/16 | Not eligible | Scopus |
|  | Rashidi F etal ,2023 | Pelvic floor disorder and relevant factors in Iranian women of reproductive age | Excluded | Excluded by title and abstract | WN | 2024/07/05-2024/08/16 | Not eligible | Scopus |
|  | Bahiyah Abdullah M etal,2023 | Awareness of pelvic organ prolapse and attitude towards its treatment among Malaysian women | Excluded | Excluded by title and abstract | WN | 2024/07/05-2024/08/16 | Not eligible | Scopus |
|  | Szatmári É etal ,2023 | Hungarian women’s health care seeking behavior and knowledge of urinary incontinence and pelvic organ prolapse | Excluded | Excluded by title and abstract | WN | 2024/07/05-2024/08/16 | Not eligible | Scopus |
|  | Chen L etal ,2023 | A qualitative study of perceptions regarding pelvic floor muscle training among pregnant women with urinary incontinence | Excluded | Excluded by title and abstract | WN | 2024/07/05-2024/08/16 | Not eligible | Scopus |
|  | Sun S etal ,2023 | An evaluation of the effects of gestational weight gain on the early postpartum pelvic floor using Transperineal ultrasound | Excluded | Excluded by title and abstract | WN | 2024/07/05-2024/08/16 | Not eligible | Scopus |
|  | Da Costa Souza H etal,2023 | Prevalence of pelvic floor disorders and the associated quality of life among institutionalized and noninstitutionalized elderly women | Excluded | Excluded by title and abstract | WN | 2024/07/05-2024/08/16 | Not eligible | Scopus |
|  | Serin AN etal ,2023 | A serious consequence of pelvic organ prolapse | Excluded | Excluded by title and abstract | WN | 2024/07/05-2024/08/16 | Not eligible | Scopus |
|  | Dickstein DR etal , 2023 | Sexual health and treatment-related sexual dysfunction in sexual and gender minorities with prostate cancer | Excluded | Excluded by title and abstract | WN | 2024/07/05-2024/08/16 | Not eligible | Scopus |
|  | Gari AM etal ,2023 | Prevalence of stress urinary incontinence and risk factors among Saudi females | Excluded | Excluded by title and abstract | WN | 2024/07/05-2024/08/16 | Not eligible | Scopus |
|  | Gupta B etal ,2023 | Changes in pelvic floor symptoms after procedural interventions for uterine leiomyomas: a systematic review | Excluded | Excluded by title and abstract | WN | 2024/07/05-2024/08/16 | Not eligible | Scopus |
|  | Peinado Molina RA etal ,2023 | Influence of pelvic floor disorders on quality of life in women | Excluded | Excluded by title and abstract | WN | 2024/07/05-2024/08/16 | Not eligible | Scopus |
|  | Hambisa, H.D etal ,2023 | Magnitude of symptomatic pelvic floor dysfunction and associated factors amongst women in Western Ethiopia | Excluded | Duplication | WN | 2024/07/05-2024/08/16 | Eligible | Scopus |
|  | Byamugisha J etal ,2023 | Characteristics and outcomes of patients with pelvic organ prolapse | Excluded | Excluded by title and abstract | WN | 2024/07/05-2024/08/16 | Not eligible | Scopus |
|  | Chi X etal ,2023 | Influence of different obstetric factors on early postpartum pelvic floor function in primiparas after vaginal delivery | Excluded | Excluded by title and abstract | WN | 2024/07/05-2024/08/16 | Not eligible | Scopus |
|  | Sawai M etal ,2022 | Prevalence of symptoms of pelvic floor dysfunction and related factors among Japanese female healthcare workers | Excluded | Excluded by title and abstract | WN | 2024/07/05-2024/08/16 | Not eligible | Scopus |
|  | Meierhofer R etal,2022 | Water carrying in hills of Nepal–associations with women’s musculoskeletal disorders, uterine prolapse, and spontaneous abortions | Excluded | Excluded by title and abstract | WN | 2024/07/05-2024/08/16 | Not eligible | Scopus |
|  | Ansari MK etal,2022 | Pelvic organ prolapse in perimenopausal and menopausal women | Excluded | Excluded by title and abstract | WN | 2024/07/05-2024/08/16 | Not eligible | Scopus |
|  | Saudi RA etal ,2022 | Prevalence and the degree of distress of pelvic floor disorders symptoms in women seeking primary health care at Ismailia governorate | Excluded | Excluded by title and abstract | WN | 2024/07/05-2024/08/16 | Not eligible | Scopus |
|  | Tugume R etal ,2022 | Pelvic organ prolapse and its associated factors among women attending the gynecology outpatient clinic at a tertiary hospital in Southwestern Uganda | Excluded | Excluded by title and abstract | WN | 2024/07/05-2024/08/16 | Not eligible | Scopus |
|  | Maroyi R etal ,2021 | Prevalence of urinary incontinence in pregnant and postpartum women in the Democratic Republic of Congo | Excluded | Excluded by title and abstract | WN | 2024/07/05-2024/08/16 | Not eligible | Scopus |
|  | Borsamo A etal,2021 | Factors associated with delay in seeking treatment among women with pelvic organ prolapse at selected general and referral hospitals of Southern Ethiopia | Excluded | Excluded by title and abstract | WN | 2024/07/05-2024/08/16 | Not eligible | Scopus |
|  | Beketie ED etal,2021 | Symptomatic pelvic floor disorders and its associated factors in South-Central Ethiopia | Excluded | Duplication | WN | 2024/07/05-2024/08/16 | Eligible | Hanari |
|  | Assefa Demissie B etal,2024 | Prevalence and associated factors of symptomatic pelvic floor disorders among women living in Debre Tabor Town, Northwest Amhara, Ethiopia | excluded | Duplication | WN | 2024/07/05-2024/08/16 | Eligible | Hanari |
|  | Kebede BN etal,2021 | Prevalence of pelvic floor disorder and associated factors among women in Arba Minch Health and Demographic surveillance site, Gamo Zone, Southern Ethiopia | Excluded | Duplication | WN | 2024/07/05-2024/08/16 | Eligible | Hanari |
|  | Dheresa M etal,2019 | Increasing trends of under-five mortality in the aftermath of Millennium Development Goal in Eastern Ethiopia | Excluded | Duplication | WN | 2024/07/05-2024/08/16 | Eligible | Hanari |
|  | Dheresa M, etal, 2020 | Women’s health seeking behavior for pelvic floor disorders and its associated factors in eastern Ethiopia | Excluded | Excluded by title and abstract | WN | 2024/07/05-2024/08/16 | Not eligible | Hanari |
|  | Megabiaw B etal,2013 | Pelvic floor disorders among women in Dabat district, northwest Ethiopia | Excluded | Duplication | WN | 2024/07/05-2024/08/16 | Eligible | Hanari |
|  | Hambisa HD etal,2023 | Magnitude of symptomatic pelvic floor dysfunction and associated factors amongst women in Western Ethiopia | Excluded | Duplication | WN | 2024/07/05-2024/08/16 | Eligible | Hanari |
|  | Beketie ED etal,2021 | Symptomatic pelvic floor disorders and its associated factors in South-Central Ethiopia | Excluded | Duplication | WN | 2024/07/05-2024/08/16 | Eligible | Hanari |
|  | Gedefaw G, etal,2020 | Effect of cesarean section on initiation of breast feeding | Excluded | Excluded by title and abstract | WN | 2024/07/05-2024/08/16 | Not eligible | Hanari |
|  | Zeleke BM etal, 2013 | Depression among women with obstetric fistula, and pelvic organ prolapse in northwest Ethiopia | Excluded | Excluded by title and abstract | WN | 2024/07/05-2024/08/16 | Not eligible | Hanari |
|  | Dheresa M etal,2018 | One in five women suffer from pelvic floor disorders in Kersa district Eastern Ethiopia | Excluded | Duplication | WN | 2024/07/05-2024/08/16 | Eligible | Hanari |
|  | Borsamo A etal,2023 | Associated factors of pelvic organ prolapse among patients at Public Hospitals of Southern Ethiopia | Excluded | Excluded by title and abstract | WN | 2024/07/05-2024/08/16 | Not eligible | Hanari |
|  | Siyoum M etal, 2024 | Prevalence and risk factors of pelvic organ prolapse among women in Sidama region, Ethiopia | Excluded | Excluded by title and abstract | WN | 2024/07/05-2024/08/16 | Not eligible | Hanari |
|  | Benti Terefe A etal,2022 | Factors associated with nursing student satisfaction with their clinical learning environment at wolkite university in southwest Ethiopia | Excluded | Excluded by title and abstract | WN | 2024/07/05-2024/08/16 | Not eligible | Hanari |
|  | Belayneh T etal,2020 | Pelvic organ prolapse in Northwest Ethiopia: a population-based study | Excluded | Excluded by title and abstract | WN | 2024/07/05-2024/08/16 | Not eligible | Hanari |
|  | Ali A etal,2022 | Prostate zones and cancer: lost in transition | Excluded | Excluded by title and abstract | WN | 2024/07/05-2024/08/16 | Not eligible | Hanari |
|  | Masenga GG etal,2018 | Prevalence and risk factors for pelvic organ prolapse in Kilimanjaro, Tanzania | Excluded | Excluded by title and abstract | WN | 2024/07/05-2024/08/16 | Not eligible | Hanari |
|  | Zeleke BM etal,2017 | Vasomotor symptoms are associated with depressive symptoms in community-dwelling older women | Excluded | Excluded by title and abstract | WN | 2024/07/05-2024/08/16 | Not eligible | Hanari |
|  | Obsa MS etal,2022 | Determinants of dyslipidemia in Africa: a systematic review and meta-analysis | Excluded | Excluded by title and abstract | WN | 2024/07/05-2024/08/16 | Not eligible | Hanari |
|  | Zeleke BM etal,2013 | Depression among women with obstetric fistula, and pelvic organ prolapse in northwest Ethiopia | Excluded | Excluded by title and abstract | WN | 2024/07/05-2024/08/16 | Not eligible | Hanari |
|  | Etana G etal,2021 | Prevalence of work related musculoskeletal disorders and associated factors among bank staff in Jimma city, Southwest Ethiopia | Excluded | Excluded by title and abstract | WN | 2024/07/05-2024/08/16 | Not eligible | Hanari |
|  | Dirriba AB etal,2021 | Prevalence of anxiety disorder and associated factors among voluntary counseling and HIV testing clients at governmental health centers in 2017 in Addis Ababa, Ethiopia | Excluded | Excluded by title and abstract | WN | 2024/07/05-2024/08/16 | Not eligible | Hanari |
|  | Kebede BN etal,2021 | Prevalence of pelvic floor disorder and associated factors among women in Arba Minch Health and Demographic surveillance site, Gamo Zone, Southern Ethiopia | Excluded | Duplication | TB | 2024/07/05-2024/08/16 | eligible | DOAJ |
|  | Gedefaw G, and Demis etal,2020 | Burden of pelvic organ prolapse in Ethiopia: a systematic review and meta-analysis | Excluded | Excluded by title and abstract | TB | 2024/07/05-2024/08/16 | Not eligible | Others/google |
|  | Mengistu Z,2023 | pelvic floor disorders: learning from chronicity & chronic care models | Excluded | Excluded by result not reported | TB | 2024/07/05-2024/08/16 | Not eligible | google |
