# Supplementary material for: Prevalence and associated factors affecting pelvic floor disorder among women in Ethiopia: A systematic review and meta-analysis
Source: PLoS One. 2025 Jul 18;20(7):e0328184. doi: 10.1371/journal.pone.0328184 (PMC12273982; doi:10.1371/journal.pone.0328184)
Supplement: S1 File — (DOCX) [file pone.0328184.s003.docx]

S1 File. Search strategies for different databases.

For PubMed search (((prevalence OR epidemiology OR magnitude) AND (pelvic floor disorder OR pelvic floor defect OR pelvic floor dysfunctions)) AND (associated factors OR determinate factors OR risk factors)) AND (Ethiopia), For Scopus TITLE-ABS-KEY ( ( ( prevalence OR epidemiology OR magnitude ) AND ( pelvic AND floor AND disorder OR pelvic AND floor AND defect OR pelvic AND floor AND dysfunctions ) ) AND ( associated AND factors OR determinate AND factors OR risk AND factors ) ) AND ( Ethiopia ) AND PUBYEAR > 2013 AND PUBYEAR < 2024 AND ( LIMIT-TO ( LANGUAGE , "English" ) ) AND ( LIMIT-TO ( DOCTYPE , "ar" ) ),For Hinari<title>"(prevalence OR epidemiology OR magnitude) AND (pelvic floor disorder OR pelvic floor defect OR pelvic floor dysfunctions) AND (associated factors OR determinate factors OR risk factors) AND (Ethiopia)"</title>
